# Heavy strength training effects on physiological determinants of endurance cyclist performance: a systematic review with meta-analysis

**DOI:** 10.1007/s00421-025-05883-2

**Published:** 2025-07-09

**Authors:** Cristian Llanos-Lagos, Rodrigo Ramirez-Campillo, Eduardo Sáez de Villarreal

**Affiliations:** 1 Physical Performance Sports Research Centre (PPSRC), Universidad Pablo de Olavide, 41704 Seville, Spain; 2 Exercise and Rehabilitation Sciences Institute. School of Physical Therapy. Faculty of Rehabilitation Sciences, Universidad Andres Bello, 7591538 Santiago, Chile; 3 Laboratorios de Ciencias del Deporte y Rendimiento Humano, Instituto de Alta Investigación, Universidad de Tarapacá, Casilla 7D, Arica, Chile; 4 Department of Physical Activity Sciences, Universidad de Los Lagos, Osorno, Chile

**Keywords:** Endurance cycling, Resistance training, Concurrent training, Cycling efficiency

## Abstract

**Background:**

Endurance cycling performance is determined by maximal oxygen uptake (VO_2_max), maximal metabolic steady state (MMSS), non-oxidative energy contribution (i.e., anaerobic capacity and anaerobic power) and cycling efficiency and power related to VO_2_max (pVO_2_max). Strength training can improve these variables. However, is yet to be clarified the effects of heavy strength training (≥ 80% of one repetition maximum).

**Aim:**

The aim of this systematic review with meta-analysis was to analyse heavy strength training effects on physiological determinants of endurance cyclists’ performance.

**Methods:**

A systematic search was carried out in PubMed, Web of Science and Scopus including articles indexed up to February 2025. Following the PICOS criteria: Population, endurance cyclists aged ≥ 18 years or older, without restriction of sex or performance level; Intervention, heavy strength training (≥ 3 weeks); Comparator, group that performed cycling endurance training without receiving heavy strength training; Outcome, physiological determinants of endurance cycling (i.e., VO_2_max, pVO_2_max, MMSS, cycling efficiency, anaerobic capacity, and anaerobic power) and/or cycling performance (i.e., time to exhaustion and time trial [combined for analyses]), measured before and after the intervention and; Study design, randomised and non-randomised controlled studies. Risk of bias in studies was assessed (PEDro), and certainty of evidence at the outcome level (GRADE). Random-effects meta-analyses (for VO_2_max, pVO_2_max, MMSS, anaerobic capacity, anaerobic power and cycling performance), three-level random-effects meta-analyses (for cycling efficiency) and moderator analyses (i.e., participant and intervention characteristics) were conducted. Significance was set as *p* ≤ 0.05.

**Results:**

Included studies (n = 17) comprised 262 participants (60 female) with a mean initial VO_2_max level of 61.25 ml/kg/min, with interventions lasting between 5 and 25 weeks, with 1–3 sessions per week. Compared to controls, heavy strength training showed a significant effect on cycling efficiency (effect size [ES] = 0.353, *p* = 0.012, LRT_level2; level3_ = 1), anaerobic power (ES = 0.560, *p* = 0.024, *I*^2^ = 29.100) and cycling performance (ES = 0.463, *p* = 0.016, *I*^2^ < 0.001), with no significant effect on VO_2_max, pVO_2_max, MMSS, and anaerobic capacity (all *p* ≥ 0.263, *I*^2^ < 0.001). No significant moderating effect was found for participant characteristics (i.e., sex, body mass, height, performance level, and strength training experience) or intervention characteristics (i.e., duration, training frequency, total sessions) (all *p* ≥ 0.170). Results presented low certainty of evidence.

**Conclusion:**

Heavy strength training can improve cycling performance (i.e., time to exhaustion; time trial) in endurance cyclist. This improvement may be mainly due to an improvement in cycling efficiency and anaerobic power. These results occur without changes in VO_2_max, pVO_2_max, MMSS or anaerobic capacity. Nonetheless, the low certainty of evidence precludes robust recommendations regarding optimal implementation of heavy strength training.

**Protocol registration:**

The original protocol was registered (https://osf.io/75xt4) at the Open Science Framework.

**Graphical abstract:**

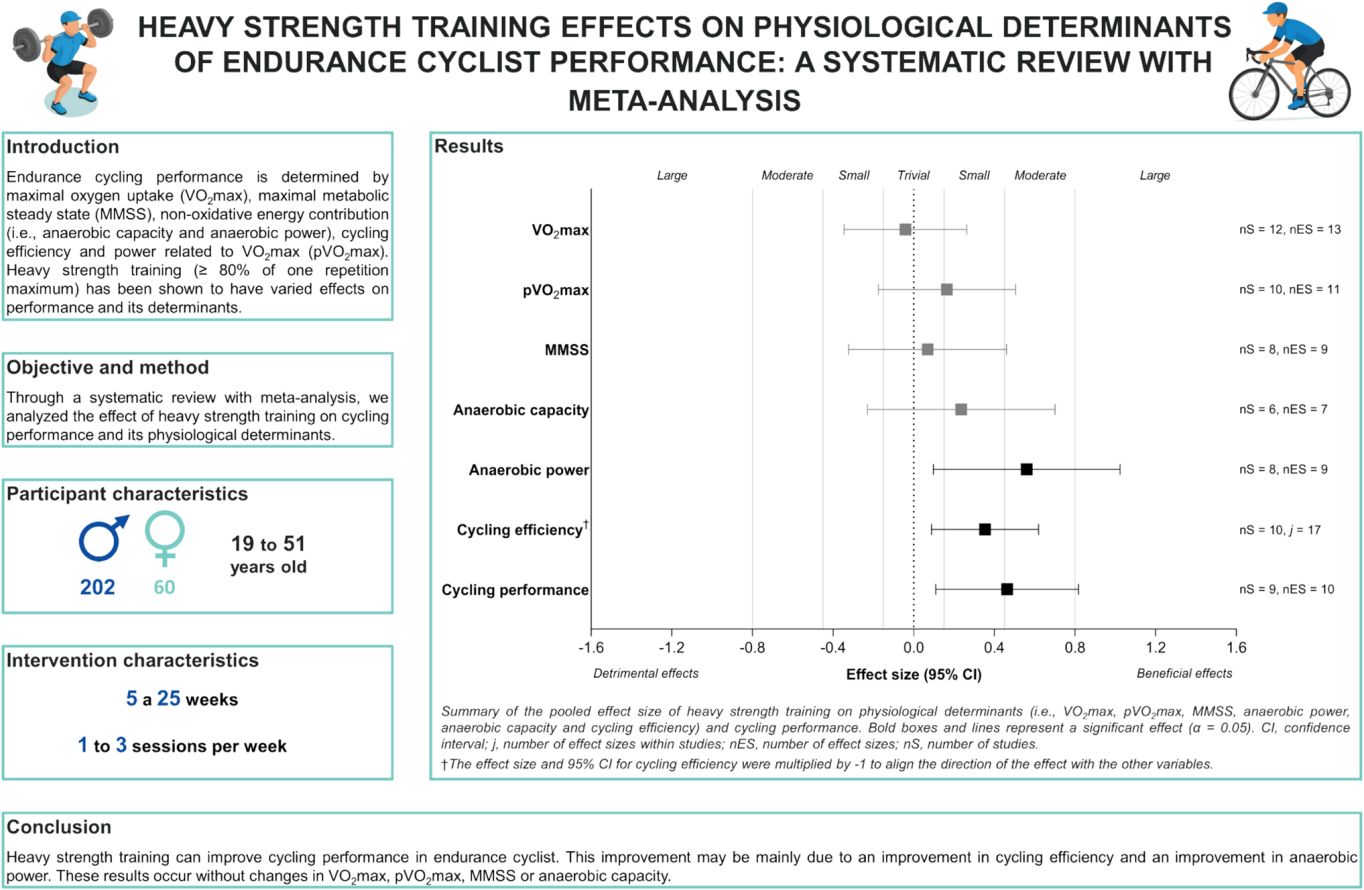

**Supplementary Information:**

The online version contains supplementary material available at 10.1007/s00421-025-05883-2.

## Introduction

The performance in endurance events such as cycling is determined by different physiological factors, such as maximal oxygen uptake (VO_2_max), maximal metabolic steady state (MMSS), anaerobic performance (i.e., non-oxidative energy contribution) and cycling efficiency (Joyner and Coyle 2008). In addition, power related to VO_2_max (pVO_2_max) has been used as a marker of the interactive contribution between VO_2_max, cycling efficiency and anaerobic performance (Jones and Carter 2000). Traditionally, these determinants of cycling performance have been addressed through aerobic and interval training performed on the bike (Faria et al. 2005). However, concurrent training, which combines strength training and endurance training in the same training program (Wilson et al. 2012), has proven to be an effective strategy to improve performance in endurance sports (Rønnestad and Mujika 2014; Lum and Barbosa 2019; Ambrosini et al. 2021; Llanos-Lagos et al. 2024a). Nevertheless, studies have shown some discrepancies in terms of performance enhancement and its determinants in cyclist (Rønnestad and Mujika 2014).

The physiological factors that determine performance can be divided into metabolic factors (i.e., VO_2_max, MMSS, cycling efficiency and anaerobic performance) and non-metabolic factors (i.e., cycling efficiency and anaerobic performance) (Paavolainen et al. 1999; Hayes and Gordon 2021). Regarding metabolic factors, VO_2_max establishes the upper limit of the oxygen transport and utilisation system during severe exercise intensity (Poole and Jones 2017), while the MMSS establishes the boundary between heavy and severe exercise intensity domains (Wasserman and McIlroy 1964; Poole et al. 2021) and depending on whether it is over or under the MMSS may have implications for muscle fatigue and exercise tolerance (Jones et al. 2010). Both physiological factors have been significantly correlated with cycling performance (Coyle et al. 1991; Bentley et al. 2001; Amann et al. 2006; Borszcz et al. 2018). However, although VO_2_max is a prerequisite for elite endurance cyclists (Faria et al. 2005), it has been shown that among subjects with similar VO_2_max, those with a higher MMSS (i.e., % VO_2_max at lactate threshold) achieve better endurance performance (Coyle et al. 1991). Whereas cycling efficiency, commonly defined as the ratio of power output to energy input (Ettema and Lorås 2009), and measured by indices such as gross efficiency, delta efficiency, work efficiency, or cycling economy (Matomäki et al. 2019), has shown that in athletes with similar VO_2_max and MMSS, those with higher cycling efficiency performed better in a 1-h cycling performance test (Horowitz et al. 1994). Cycling efficiency might be affected by metabolic and non-metabolic factors (Jobson et al. 2012). On the other hand, non-oxidative energy contribution (i.e. phosphocreatine, glycolysis and lactate formation) is provided when intensity exceeds MMSS and oxidative metabolism (i.e., fat and carbohydrate oxidation) is not sufficient to supply energy turnover, leading to increased energy production from non-oxidative metabolism (Poole et al. 2021). These efforts above the MMSS are used by cyclists during a race and are important for race performance, such as positioning at the start of the race, during the climb, overtaking other cyclists and/or sprinting at the finish line (Faria et al. 2005). For this purpose, the Wingate test or similar sprint tests are traditionally used to assess peak power output (i.e., anaerobic power) and mean power output (i.e., anaerobic capacity), to reflect the predominance of energy contribution from phosphocreatine and glycolysis and lactate formation, respectively (Bar-Or 1987; Bogdanis et al. 1998; Beneke et al. 2002). Therefore, while it has been postulated that strength training may improve cycling performance (Yamamoto et al. 2010; Aagaard and Andersen 2010; Rønnestad and Mujika 2014), it is necessary to understand by which of these physiological determinants this improvement might be produced.

Strength training can have different purposes depending on how different variables (e.g., loading, training volume, or rest intervals) are manipulated (Kraemer and Ratamess 2004). Among them, the load (often expressed as a percentage of one repetition maximum [1RM]) is possibly one of the most relevant variables. Particularly, when heavy strength training method (i.e., ≥ 80% 1RM) is implemented, improvements in different physiological determinants of performance in endurance cyclists have been produced (Sunde et al. 2010; Rønnestad et al. 2015; Vikmoen et al. 2016). This training method is mainly related to neuromuscular and morphological adaptations (Aagaard et al. 2002; Aagaard 2003; Hughes et al. 2018) thus it is expected to have an effect mainly on non-metabolic determinants (Blagrove et al. 2018; Llanos-Lagos et al. 2024a). Some results support this argument by showing that the inclusion of heavy strength training has improved cycling efficiency (Sunde et al. 2010; Vikmoen et al. 2016) and anaerobic power (Sitko et al. 2024) in cyclists. Conversely, improvements in metabolic determinants of performance have been reported in endurance athletes, including VO_2_max (Rønnestad et al. 2010a), MMSS (i.e., power at 4 mmol L^−1^ blood lactate [BLa]) (Rønnestad et al. 2015), and anaerobic capacity (Vikmoen et al. 2016). However, other studies found no favouring effect after heavy strength training (Aagaard et al. 2011; Rønnestad et al. 2015). These differences may be related to the characteristics of the participants and the intervention. For example, it has been suggested that the absence of improvement in cycling efficiency after heavy strength training might be since this type of stimulus is not sufficient to produce improvement in well-trained cyclists (Rønnestad et al. 2010a; Aagaard et al. 2011). Additionally, strength training may induce different adaptations in cyclists of different ages (Louis et al. 2012; Del Vecchio et al. 2019) and sex groups (Vikmoen and Rønnestad 2021). Concerning the intervention characteristics, it has been reported that a longer intervention duration increases the effect on running economy in runners (Denadai et al. 2017). In addition, Rønnestad et al. (2010a) reported that two sessions per week for 13 weeks improved cycling performance accompanied by an increase in muscle strength and thigh cross-sectional area (CSA), and these improvements were maintained with one session per week for two weeks. Accordingly, a more comprehensive analysis is needed to understand the effect of heavy strength training on physiological determinants and performance in endurance cyclists.

Some reviews and meta-analyses attempted to establish the effect of strength training on some physiological determinants and performance in endurance athletes (Yamamoto et al. 2010; Aagaard and Andersen 2010; Rønnestad and Mujika 2014; Lum and Barbosa 2019; Ambrosini et al. 2021). In particular, two systematic reviews with meta-analyses have focused their analyses on the effect of strength training on performance including different endurance sports (e.g., cycling, running, swimming, rowing) (Lum and Barbosa 2019; Ambrosini et al. 2021), but these studies do not consider the differences that could be present between different sport modalities, such as the difference in muscle activity and its effect on work economy (Bijker et al. 2002). Whereas to date only reviews and original research have been conducted specifically on endurance cyclists (Yamamoto et al. 2010; Aagaard and Andersen 2010; Rønnestad and Mujika 2014). This underlines the importance of a systematic review with meta-analysis focusing specifically on endurance cyclists, which updates and clarifies the body of knowledge on the effects of heavy strength training in this population. Therefore, the aim of this systematic review with meta-analysis was to analyse heavy strength training effects on physiological determinants (i.e., VO_2_max, pVO_2_max, MMSS, cycling efficiency, and non-oxidative energy contribution [anaerobic capacity and anaerobic power]) and cycling performance (i.e., time to exhaustion and time trial).

## Methods

### Experimental approach to the problem

The PRISMA guidelines (Page et al. 2021) were followed to report in a transparent manner the systematic review, the methods used and the findings. The protocol was registered on the Open Science Framework after the data analysis (https://osf.io/75xt4).

### Information sources and search strategy

The search for articles covered PubMed (in all databases), Web of Science (in all databases) and Scopus databases, and articles indexed up to August 2023 were considered for selection. Search terms and Boolean operators were used (Table SM1 of the supplementary material [SM]). No restrictions on study design, date, language, age, or sex were applied. The search was also updated in February 2025, considering notifications of new studies identified through the search strategy in the different databases. Additionally, the reference lists of includable articles, and from reviews, systematic reviews and meta-analyses retrieved from our search, were scanned for additional articles of interest.

In addition, we conducted an examination of the reference lists of eligible articles, reviews, systematic reviews, and meta-analyses retrieved from our search strategy, looking for additional articles of interest.

### Selection process

An independent reviewer (LL) examined all titles and abstracts obtained from the database searches. Articles potentially meeting the inclusion criteria (Table 1) were subjected to full-text analysis. At the time of data cross-checking, controversial cases were reviewed by two authors (SV and RRC).

**Table 1:** Inclusion and exclusion criteria for systematic review and meta-analysis

Category	Inclusion criteria	Exclusion criteria
Population	Amateur and competitive endurance cyclist (i.e., road, gravel, mountain bikers), aged ≥ 18 years old, without restriction to sex or training/competitive level	Subjects with injuries, comorbidities, or non-cycling endurance athletes
Intervention	Heavy strength training (≥ 80% 1RM) that was implemented in addition to or a partial substitute (i.e., load-matched training) for endurance cycling training, with a minimum duration of 3 weeks and including a minimum of one session per week	The programme includes alternative methods in addition to strength training (e.g., body vibration or electrical stimulation), and/or supplementations (e.g., creatin)
Comparator	A control group engaged in endurance cycling training, either without receiving strength training or receiving it with light loads (< 40% 1RM or > 20RM)	Absence of control group
Outcome	VO_2_max, pVO_2_max, MMSS, cycling efficiency, anaerobic capacity, anaerobic power, and/or cycling performance were recorded at least once before and after the strength training intervention	Baseline and/or follow-up data not available
Study design	Randomised and non-randomized controlled studies	Cross-sectional, observational, or case studies

*1RM* one repetition maximum; *MMSS* maximal metabolic steady state; *RM* repetition maximum; *VO*_*2*_*max* maximal oxygen uptake; *pVO*_*2*_*max* power related to VO_2_max

### Eligibility criteria

Studies were considered eligible for inclusion based on the P.I.C.O.S. criteria (Participants, Intervention, Comparator, Outcome and Study Design), as detailed in Table 1.

### Data collection process

The mean and standard deviation (SD) data for participant characteristics, intervention and main outcomes of the included studies were collected by one researcher (LL). When the values were presented in figure data only, these were extracted using the validated (Drevon et al. 2017) WebPlotDigitizer software (version 4.6, Pacifica, California, USA). In the case of controversial data, these were discussed by three reviewers (LL, RRC and SV).

#### Participants

Participant characteristics were registered, including sex (male, female mixed samples), age (years), body mass (kg), height (cm), performance level (i.e., initial VO_2_max), and strength training experience (yes, no, not reported). Participants in this study had to be 18 years of age or older to ensure that they have completed puberty, a period marked by significant hormonal changes that can impact response to strength training (Goswami et al. 2014). The performance level was determined by the initial VO_2_max (mL kg^−1^ min^−1^). Systematic strength training experience was recorded according to the criteria reported in each study.

#### Strength training intervention

Characteristics of the strength training intervention were collected, such as duration (weeks), training frequency (sessions per week) and total number of sessions. Following a classification of strength training methods according to training objective and training load (i.e., %1RM) used in reviews related to endurance sports (Beattie et al. 2014; Blagrove et al. 2018), heavy strength training was considered when lower body exercises (e.g., barbell squat, leg press) were performed with a load ≥ 80% of 1RM or ≤ 7 RM or the intensity of maximal strength training was expressed. Groups that, in addition to heavy strength training, applied other strength training methods, such as strength training with submaximal load (40–79% 1RM or 8–20 RM), or plyometric training (e.g., squat with jumps, drop jump) were also considered. The control group was those who did not implement strength training or implemented strength training with low loads (i.e., ≤ 40% 1RM or ≥ 20 RM).

#### Outcome measurements

Pre and post-intervention values of VO_2_max, pVO_2_max, MMSS, cycling efficiency, anaerobic performance, and cycling performance were collected. MMSS was considered if it was measured maximal lactate steady state, second lactate threshold, lactate turn point, respiratory compensation point, onset of blood lactate accumulation, second ventilatory threshold or critical power. Cycling efficiency was considered if it was measured as i) gross efficiency, the ratio of external work to total energy expenditure, ii) net efficiency, the ratio of external work to the difference between total energy expenditure and resting energy expenditure, iii) work efficiency, the ratio of external work to the difference between total energy expenditure and the energy expenditure when cycling with zero load, iv) delta efficiency, the ratio of the change in external work to the change in total energy expenditure, or v) cycling economy, the oxygen cost at a given power output or the ratio of VO_2_ to power output. The test had to be performed at submaximal intensity (i.e., at an intensity at ≤ MMSS) to ensure that the energy contribution was predominantly from oxidative metabolism, given the limitations in calculating the energy contribution from non-oxidative pathways during the assessment of cycling efficiency (MacDougall et al. 2022). The test was considered submaximal intensity when it met one or more of the following criteria: i) intensity was ≤ MMSS, ii) respiratory exchange ratio ≤ 1, iii) BLa concentrations at ≤ 4 mmol L^−1^ and/or iv) power intensity was ≤ 83% of VO_2_max (Kipp et al. 2018; Iannetta et al. 2020). Non-oxidative energy contribution was considered as those tests of maximum intensity performed on cycle ergometers (e.g., Wingate test, sprint test) or cycling track (e.g., sprint test), in efforts in which non-oxidative energy contribution is predominant (i.e., < 75 s) (Gastin 2001). Anaerobic power was considered when peak power output was recorded, while anaerobic capacity was considered when mean power output was recorded (Bar-Or 1987; Bogdanis et al. 1998). Cycling performance was recorded as cycling efforts in time trials or time until exhaustion greater than 75 s (Gastin 2001).

If a study included several tests for the same variable (e.g., time trial and time to exhaustion) and/or more than one unit of measurement was included for the same variable (e.g., W and W kg^−1^), the test and/or unit of measurement most similar to those in other studies was chosen. In cases where test measurements were reported at multiple time points (i.e., more than two time points), the initial record and the final record immediately following the intervention were selected.

### Risk of bias, risk of publication bias and certainty assessment

We assessed the risk of bias of the studies using the 11-item Physiotherapy Evidence Database scale (PEDro scale) (Maher et al. 2003; de Morton 2009). However, in the context of our systematic review, we omitted items 5–7 due to the usual lack of blinding of researchers, assessors, and subjects in physical activity interventions (de Morton 2009; González-Mohíno et al. 2020). Consequently, the scale was set to a maximum rating of 7 points. According to previous criteria (González-Mohíno et al. 2020), the studies were categorized as follows: ≥ 6 points = “low risk”, 4–5 points = “moderate risk”, and ≤ 3 points = “high risk”.

The risk of publication bias was assessed using the funnel plot for each main outcome. The presence of risk of publication bias was considered when asymmetry was identified in the funnel plot in combination with the Egger's test (i.e., *p* ≤ 0.05) (Egger et al. 1997; Fernández-Castilla et al. 2021). To analyse the certainty of evidence, we used the Grading of Recommendations Assessment, Development and Evaluation (GRADE) approach (Guyatt et al. 2011; Zhang et al. 2019a, b). A high certainty of evidence was established at the outset, which was downgraded according to the next criteria: risk of bias, downgraded by one or two levels if the risk of bias was moderate (4–5 points) or high (≤ 3 points), respectively; inconsistency, downgraded by one level if significant heterogeneity was found (i.e., *p* ≤ 0.05) in the Cochrane *Q*-test; indirectness, not downgraded as indirectness was warranted in the PICOS criteria; imprecision, downgraded by one level if imprecision occurred when the number of control and experimental participants was < 800 or if the confidence interval crossed the small effect size threshold (i.e., ES = − 0.15–0.15); risk of publication bias, downgraded by one level if the funnel plot was observed to be asymmetric in the Egger’s test (i.e., *p* ≤ 0.05).

### Effect measures

Mean, SD, and sample size (N) values were collected for the control and experimental groups before and after the intervention. When the variation of the data was reported as standard error (SE), the SD was calculated as SE multiplied by the square root of N (Higgins et al. 2019). The mean change and pooled post standard deviation were calculated using the following formulae (Becker 1988; Morris and DeShon 2002):

1 $${\mathrm{M}}_{{{\mathrm{change}},{\text{ experimental}}}} = {\mathrm{M}}_{{{\mathrm{post}},{\text{ experimental}}}} - {\mathrm{M}}_{{{\mathrm{pre}},{\text{ experimental}}}}$$

and

2 $${\mathrm{M}}_{{{\mathrm{change}},{\text{ control}}}} = {\mathrm{M}}_{{{\mathrm{post}},{\text{ control}}}} - {\mathrm{M}}_{{{\mathrm{pre}},{\text{ control}}}}$$

where M_change_ is the raw mean difference, M_pre_ and M_post_ are the means reported before and after the intervention for the experimental and control groups, respectively.

3 $${\mathrm{SD}}_{{{\mathrm{pooled}},{\text{ post}}}} = \sqrt {\frac{{\left( {n_{1} - 1} \right)SD_{1}^{2} + \left( {n_{2} - 1} \right)SD_{2}^{2} }}{{n_{1} + n_{2} - 2}}}$$

where SD_1_ and SD_2_ represent the standard deviations of the experimental and control groups, respectively, and n_1_ and n_2_ denote the sample sizes of the experimental and control groups, respectively.

These data were used to calculate effect sizes as Hedges' g (ES) (Hedges and Olkin 1985), adjusted for small sample sizes (Borenstein et al. 2009) as follows:

4 $${\mathrm{ES}} = { }\frac{{\left( {{\mathrm{M}}_{{{\mathrm{change}},{\text{ experimental}}}} - {\mathrm{M}}_{{{\mathrm{change}},{\text{ control}}}} } \right)}}{{{\mathrm{SD}}_{{{\mathrm{pooled}},{\text{ post}}}} }} \times J$$

where *J*

5 $$J = 1 - \frac{3}{{4\left( {n_{1} + n_{2} - 2} \right) - 1}}$$

The effect size thresholds were set at 0.15, 0.45 and 0.80, for small, moderate, and large magnitude, respectively (Swinton et al. 2022).

### Statistical analyses

We conducted a meta-analysis for each of the main outcomes (VO_2_max, pVO_2_max, MMSS, anaerobic capacity, anaerobic power and cycling performance). A randomized effects model was used to deal with the sources of variation between studies (e.g., participant and intervention characteristics). Whereas in the case of cycling efficiency, since it is generally assessed at more than one intensity (i.e., cycling efficiency at different absolute or relative power), a three-level meta-analysis was carried out (Van den Noortgate et al. 2013; Cheung 2014) which, instead of a random effects meta-analysis model that analyses sampling (level 1) and between-study (level 3) variance (Borenstein et al. 2009), this allows to include within-study variance (level 2). Therefore, including more than one outcome within study allows to avoid selecting a “representative” ES (Park and Beretvas 2019) or averaging ESs (Higgins et al. 2019; Park and Beretvas 2019), increasing the sample size (Park and Beretvas 2019) and not overestimating the standard error (Moeyaert et al. 2017), respectively. For all analyses, the restricted maximum likelihood method was used to estimate model parameters ($\tau^{2}$) for continuous data (Veroniki et al. 2016), and the Knapp-Hartung method (Knapp and Hartung 2003) following a *t*-distribution was used to compute test statistics and confidence intervals (CI). When more than one experimental group was present in the same study, the sample size of the control group was divided by the number of experimental groups (Higgins et al. 2019).

The presence of statistical heterogeneity for each analysis (i.e., VO_2_max, pVO_2_max, MMSS, anaerobic capacity, anaerobic power and cycling performance) was determined by the significance of the heterogeneity test (*Q*-test). Because small sample sizes are common in sports science (Abt et al. 2020), the *p*-value for *Q*-test was set at 0.10 as recommended (Higgins et al. 2019). While for cycling efficiency, the one-side log-likelihood-ratio test (LRT) was used to analyse within (LRT_level2_) and between-study variance (LRT_level3_) heterogeneity (Assink and Wibbelink 2016). An outlier was identified when the upper limit of the 95% CI for an ES falls below the lower limit of the confidence interval for the pooled effect, or conversely, when the lower limit of the 95% CI for an ES exceeds the upper limit of the confidence interval for the pooled effect (Harrer et al. 2021). If an outlier was detected, a sensitivity analysis was conducted both with and without the outlier ES to evaluate its influence on the analysis (Harrer et al. 2021) (i.e., *p*-value from *Q*-test).

A moderator analysis was carried out for each analysis, using meta-regression (i.e., age, body mass, height, initial VO_2_max, weeks, sessions per week and number of total sessions) and sub-group analysis (i.e., sex). Meta-analysis of the random effects model and its graphical representations (i.e., forest plot and funnel plot) was performed using R software (version 4.4.2) with the *metafor* package (Viechtbauer 2010). A three-level meta-analysis was also performed using the same software and package. For the three-level meta-analysis, the syntax provided by Assink & Wibbelink (Assink and Wibbelink 2016) was applied, while the forest plot and funnel plot were generated using the syntax of Fernández-Castilla et al. (2020). Summary results for all pooled effect sizes were created using GraphPad Prism 10 (version 10.1.0). Statistical significance was set at *p*-value ≤ 0.05.

## Results

### Study selection

**Fig. 1:**
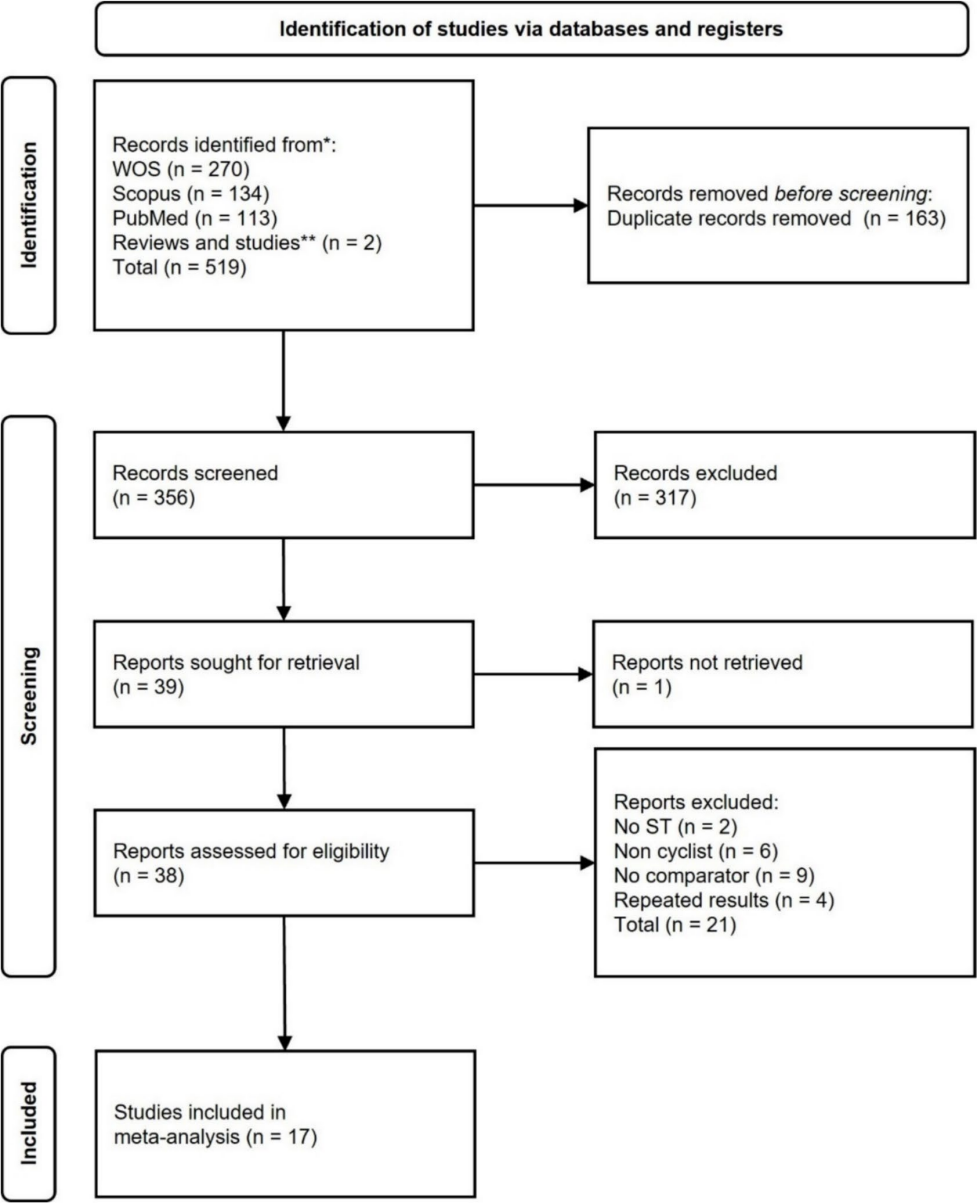
Flow diagram of the studies selection process. *Studies found in the reference lists of articles, reviews, systematic reviews, and meta-analyses retrieved from our search strategy. **Studies found from notifications of new studies found in the search strategy in the different databases

A total of 519 records were initially identified using the search strategy (Fig. 1). After removing duplicate records, unretrieved records and articles excluded based on title and/or abstract review, 38 studies were assessed for eligibility. After a thorough review of the full text of each study, 21 studies were excluded (to see the excluded studies and their reasons for exclusion, see Table SM2). In some cases, subsequent studies (Rønnestad et al. 2010b, 2016) presented new findings, so the results of those variables that had not been included in the primary research (Rønnestad et al. 2011, 2015) were included in the analysis where appropriate. As a result, 17 studies were included in the systematic review and meta-analyses.

### Study characteristics

The characteristics of the participants and details of the strength training intervention are described in Table 2, while the results for each variable analysed in the included studies are summarised in Tables 3 and 4. The VO_2_max was analysed in thirteen groups of twelve studies (Bishop et al. 1999; Jackson et al. 2007; Levin et al. 2009; Rønnestad et al. 2010a, b, 2015; Hausswirth et al. 2010; Sunde et al. 2010; Aagaard et al. 2011; Vikmoen et al. 2016; Beattie et al. 2017; Ji et al. 2022), while pVO_2_max was assessed in eleven groups of ten studies (Jackson et al. 2007; Levin et al. 2009; Rønnestad et al. 2010a, b, 2015; Hausswirth et al. 2010; Vikmoen et al. 2016; Beattie et al. 2017; Del Vecchio et al. 2019; Ji et al. 2022). The MMSS was analysed in nine groups of eight studies (Bishop et al. 1999; Hausswirth et al. 2010; Sunde et al. 2010; Aagaard et al. 2011; Rønnestad et al. 2015; Vikmoen et al. 2016; Beattie et al. 2017; Ji et al. 2022), anaerobic capacity in seven groups of six studies (Rønnestad et al. 2010a, b, 2015; Vikmoen et al. 2016; Del Vecchio et al. 2019; Ji et al. 2022) and anaerobic power in nine groups of eight studies (Rønnestad et al. 2010a, b, 2015; Vikmoen et al. 2016; Beattie et al. 2017; Del Vecchio et al. 2019; Ji et al. 2022; Sitko et al. 2024). Additionally, cycling efficiency was assessed in eleven groups of ten studies (Jackson et al. 2007; Hausswirth et al. 2010; Sunde et al. 2010; Rønnestad et al. 2010a, 2016; Aagaard et al. 2011; Vikmoen et al. 2016; Beattie et al. 2017; Luckin-Baldwin et al. 2021; Ji et al. 2022), yielding 17 effect sizes because this variable was measured at more than one intensity in some studies. Cycling performance was assessed in nine studies, involving ten groups (Bishop et al. 1999; Sunde et al. 2010; Rønnestad et al. 2010a, 2011, 2015; Aagaard et al. 2011; Vikmoen et al. 2016; Ji et al. 2022; Sitko et al. 2024). The analysis included a total of 262 participants, 145 in the experimental group (34 females) and 117 in the control group (26 females). Their mean age was 31.28 years (range 19.50–51.45), mean weight 72.28 (range 59.80 to 80.20), mean height 178.73 (range 169.50 to 183.00), and mean initial VO_2_max 61.25 (range 48.25–75.50). Strength training interventions lasted a mean of 13.63 weeks (range 5.00–25.00 weeks), with a frequency of 2.21 sessions per week (range 1.00–3.00), totalling 27.65 sessions on average (range 15.00–48.00). The reduced number of studies for the moderator ‘systematic strength training experience’ precluded a moderator analysis.

**Table 2:** Characteristics of participants and strength training intervention from the included studies

Authors	Participant characteristics							Strength training intervention						
	G	n	Age	BM	Ht	iVO_2_max	STexp	D	Fq	TS	Exercises	Load	Sets x repetitions	Rest
Bishop, D. et al. (1999)	H	14 (F)	30	59	NR	48	No	12	2	24	Squat	4wk: 80–85% 1RM; 4wk: 85–90% 1RM; 4wk: 90–95% 1RM	4wk: 5 × 6–8; 4wk: 4 × 4–6; 4wk: 3 × 2–4	3 min
	C	7 (F)	30	60	NR	48	No							
Jackson, N. et al. (2007)	H	9 (M = 8; F = 1)	31	NR	NR	48	No	10	3	30	Squat, machine leg curls, machine leg press, machine single-leg step-ups	1wk: 50% 1RM; 9wk: 85% 1RM	1wk: 2 × 10; 9wk: 4 × 4	2 min
	C	5 (M = 3; F = 2)	27	NR	NR	55	No							
Levin, G. et al. (2009)	H + SL + PL	7 (M)	25	79	181	62	No	6	3	18	H: Lunges, squats, straight-leg deadlift, seated calf raises. SL: Single-leg calf raise, single-leg press, knee extension, knee flexion, standing calf raises. PL: Jump squat, single-leg jump squat	*Muscle strength*, *power development* and *hypertrophy* loads	PL: 3 × 6; SL 3 × 6–12; HL: 4 × 5	2 min
	C	7 (M)	37	76	179	63	No							
Hausswirth, C. et al. (2010)	H	7 (M)	30	70	176	70	NR	5	3	15	Leg extension, leg press, hamstring curl, leg curl	87–97% 1RM	3–5 × 3–5	3 min
	C	7 (M)	32	69	175	68	NR							
Rønnestad, B. et al. (2010a)	H	6 (M)	29	NR	185	65	No	25	2	37	Half squat, single-leg leg press, single-leg hip flexion, ankle plantar flexion	3wk: 75–85% 1RM; 3wk: 80–87% 1RM; 6wk: 85–90% 1RM; 13wk: 80–85	3wk: 3 × 6–10; 3wk: 3 × 5–8; 6wk: 3 × 4–6; 13wk: 1–2 × 5–6	2 min
	C	6 (M = 5; F = 1)	31	NR	181	67	No							
Rønnestad, B. et al. (2010b)	H	11 (M)	27	76	NR	67	No	12	2	24	Half squat, single-leg leg press, single-leg hip flexion, ankle plantar flexion	3wk: 75–85% 1RM; 3wk: 80–87% 1RM; 6wk: 85–90% 1RM	3wk: 3 × 6–10; 3wk: 3 × 5–8; 6wk: 3 × 4–6	3 min
	C	9 (M = 7; F = 2)	30	75	NR	66	No							
Sunde, A. et al. (2010)	H	8 (M = 7; F = 1)	30	73	178	63	No	8	3	24	Half squat in a Smith-machine	90% 1RM	4 × 4	3 min
	C	5 (M = 3; F = 2)	36	75	178	59	No							
Aagaard, P. et al. (2011)	H	7 (M)	20	69	181	74	NR	16	3	40	Isolated knee extension, incline leg press, hamstring curls, calf raises	1wk: 67–75% 1RM; 2wk: 75–80% 1RM; 2wk: 80–85%1RM; 11wk: 85–87% 1RM	4 × 5–12	1–2 min
	C	7 (M)	20	72	181	72	NR							
Rønnestad, B. et al. (2011)	H	11(M)	27	76	NR	67	No	12	2	24	Half-squat in a Smith-machine, singe-leg leg press, single-leg hip flexion, toe raise	3wk: 75–85% 1RM; 3wk: 80–87% 1RM; 6wk: 85–90%1RM	3wk: 3 × 6–10; 3wk: 3 × 5–8; 6wk: 3 × 4–6	3 min
	C	9 (M = 7; F = 2)	30	75	NR	66	No							
Rønnestad, B. et al. (2015)	H	9 (M)	19	66	178	78	NR	25	1–2	32	Half squat, single-leg leg press, standing single-leg hip flexion, ankle plantar flexion	3wk: 75–85% 1RM; 3wk: 80–87% 1RM; 4wk: 85–90%1RM; 15wk: 75–85% 1RM	3wk: 3 × 6–10; 3wk: 3 × 5–8; 4wk: 3 × 4–6; 15wk: 3 × 5	2 min
	C	7 (M)	20	74	183	73	NR							
Rønnestad, B. et al. (2016)	H	9 (M)	19	66	178	78	NR	25	1–2	32	Half squat, single-leg leg press, single-leg hip flexion, ankle plantar flexion	3wk: 75–85% 1RM; 3wk: 80–87% 1RM; 4wk: 85–90%1RM; 15wk: 75–85% 1RM	3wk: 3 × 6–10; 3wk: 3 × 5–8; 4wk: 3 × 4–6; 15wk: 3 × 5	2 min
	C	7 (M)	20	74	183	73	NR							
Vikmoen, O. et al. (2016)	H	11 (F)	32	62	169	54	No	11	2	22	Half squat in a smith machine, single-leg leg press, single-leg hip flexion, ankle plantar flexion	3wk: 75–85% 1RM; 3wk: 80–90% 1RM; 5wk: 85–90% 1RM	3 × 4–10	NR
	C	8 (F)	35	66	170	55	No							
Beattie, K. et al. (2017)	H + SL + PL	6 (M)	38	69	177	63	NR	20	2	40	H: Trap-bar deadlift. PL: Squat jump. SL: Squat jump, trap-bar deadlift, goblet squat, romanian deadlift, split squat	*Maximal* and *explosive* strength loads	PL: 2–3 × 3–5; SL: 2–3 × 5–12; HL: 3 × 5–8	NR
	C	9 (M)	35	73	178	62	NR							
Del Vecchio, et al. (2019)	H + PL + SL	9 (M)	54	82	180	NR	NR	12	2	24	H: Single-leg leg press, seated hip flexion. PL: Ankle hops, side to side ankle hops, standing jump and reach, front box jump, jump from box, lateral box jump, alternating step push offs, single-leg box push offs, squat depth jumps. SL: Leg press, seated hip flexion, leg curls, leg extensions, seated calve raise standing, single-leg leg press throw	HL: 70–90% 1RM; PL: BW; SL: 40–70% 1RM	PL: 1–2 × 8–15; SL: 2–12 × 2–5; HL: 3 × 3–8	2 min
	C	7 (M)	49	79	180	NR	NR							
Luckin-Baldwin, K. et al. (2021)	H + SL	15 (M = 10; F = 5)	39	76	177	51	No	24	2	48	Half squat, glute hamstring raises, hip thrust, single-leg leg press, single-leg seated calf raises, hip flexion, hip abduction	12wk: ≥ 75% 1RM; 12wk: ≥ 85% 1RM	12wk: 3–4 × 8–12; 12wk: 3–5 × 1–6	NR
	C	15 (M = 12; F = 3)	36	77	176	53	No							
Ji, S. et al. (2022)	H	7 (M = 6; F = 1)	33	81	182	55	No	10	2	20	Single-leg leg press machine, single-leg leg extension machine, single-leg leg curl machine	75–90% 1RM	4 × several	2–3 min
	H	7 (M = 6; F = 1)	29	78	182	58	No	10	2	20	Leg press machine, leg extension machine, leg curl machine	75–90% 1RM	4 × several	2–3 min
	C	6 (M = 5; F = 1)	32	71	182	60	No							
Sitko et al. (2024)	H	12 (M)	28	70	179	NR	NR	12	2	24	Half squat, single-leg leg press, single-leg hip flexion, ankle plantar flexion	85% 1RM	3 × 6	3 min
	C	12 (M)	30	69	180	NR	NR							

*BM* body mass; *C* control; *D* duration (weeks); *F* female; *Fq* frequency (session/week), *G* group; *H* heavy strength training; *Ht* height (cm); *iVO*_*2*_*max* initial maximal oxygen uptake (VO_2_max; mL kg^−1^ min^−1^); *M* male; *n* sample size; *min* minutes; *NR* not reported; *PL*, plyometric training; *SL* submaximal load strength training; *STexp* strength training experience; *TS* total sessions; *wk* weeks; *1RM*, one repetition maximum

**Table 3:** Analysis of VO_2_max, pVO_2_max, maximum metabolic steady state, anaerobic capacity and anaerobic power of the included studies

Study	Participant characteristics		VO_2_max (mL kg^−1^ min^−1^)		pVO_2_max			Maximum metabolic steady state			Anaerobic capacity			Anaerobic power		
	G	n	Mean pre (SD)	Mean post (SD)	Measurement	Mean pre (SD)	Mean post (SD)	Measurement	Mean pre (SD)	Mean post (SD)	Test	Mean pre (SD)	Mean post (SD)	Test	Mean pre (SD)	Mean post (SD)
Bishop, D. et al. (1999)	H	14 (F)	48.20 (5.80)	48.40 (5.50)				LT (W)	177.70 (35.00)	183.30 (24.10)						
	C	7 (F)	48.30 (6.70)	48.40 (9.70)				LT (W)	179.10 (11.70)	179.90 (15.50)						
Jackson, N. et al. (2007)	H	9 (M = 8; F = 1)	47.90 (7.80)	49.30 (6.50)	W	305.60 (39.10)	305.60 (37.00)									
	C	5 (M = 3; F = 2)	55.30 (3.50)	58.90 (2.90)	W	315.00 (51.80)	330.00 (41.10)									
Levin, G. et al. (2009)	H + SL + PL	7 (M)	62.40 (5.40)	62.30 (3.20)	W	361.00 (36.00)	355.00 (27.00)									
	C	7 (M)	63.10 (1.80)	62.50 (2.70)	W	352.00 (39.00)	348.00 (37.00)									
Hausswirth, C. et al. (2010)	H	7 (M)	69.90 (6.30)	70.80 (5.50)	W	412.90 (28.00)	419.30 (29.60)	VT_2_ (%VO_2_max)	84.40 (6.10)	84.10 (4.40)						
	C	7 (M)	68.40 (10.70)	68.30 (10.10)	W	417.50 (51.50)	410.70 (44.80)	VT_2_ (%VO_2_max)	86.30 (4.70)	84.30 (6.10)						
Rønnestad, B. et al. (2010a)	H	6 (M)	65.20 (5.39)	73.90 (7.84)	W	420.00 (36.74)	454.00 (46.54)				WT (W kg^−1^)	10.20 (0.73)	10.20 (0.98)	WT (W kg^−1^)	18.50 (0.98)	19.90 (1.96)
	C	6 (M = 5; F = 1)	67.30 (6.61)	73.40 (7.59)	W	401.00 (90.63)	399.00 (80.83)				WT (W kg^−1^)	9.30 (1.47)	9.30 (1.71)	WT (W kg^−1^)	15.70 (2.69)	16.00 (3.92)
Rønnestad, B. et al. (2010b)	H	11 (M)			W	407.00 (33.17)	425.00 (33.17)				WT (W kg^−1^)	10.40 (0.66)	10.50 (0.66)	WT (W kg^−1^)	18.10 (1.99)	19.10 (1.99)
	C	9 (M = 7; F = 2)			W	403.00 (75.00)	411.00 (75.00)				WT (W kg^−1^)	9.60 (1.50)	9.50 (1.20)	WT (W kg^−1^)	16.20 (2.70)	16.20 (3.00)
Sunde, A. et al. (2010)	H	8 (M = 7; F = 1)	63.40 (6.00)	63.90 (5.60)				LT_2_ (W)	243.00 (44.00)	248.00 (42.00)						
	C	5 (M = 3; F = 2)	58.70 (8.80)	58.00 (10.80)				LT_2_ (W)	258.00 (74.00)	262.00 (78.00)						
Aagaard, P. et al. (2011)	H	7 (M)	73.50 (8.20)	75.00 (6.00)				4 mmol BLa^−1^ (W)	323.70 (44.70)	329.00 (34.70)						
	C	7 (M)	71.50 (6.00)	73.00 (2.30)				4 mmol BLa^−1^ (W)	305.00 (20.60)	324.00 (21.70)						
Rønnestad, B. et al. (2011)	H	11 (M)	66.80 (5.31)	69.00 (5.31)												
	C	9 (M = 7; F = 2)	65.90 (6.00)	69.80 (7.50)												
Rønnestad, B. et al. (2015)	H	9 (M)	78.00 (6.00)	80.00 (6.00)	W kg^−1^	5.96 (0.46)	6.11 (0.56)	4 mmol BLa^−1^ (W kg^−1^)	4.11 (0.45)	4.23 (0.41)	WT (W kg^−1^)	10.90 (0.90)	10.90 (1.10)	WT (W kg^−1^)	23.60 (2.90)	24.20 (3.40)
	C	7 (M)	73.00 (5.00)	75.00 (7.00)	W kg^−1^	5.81 (0.25)	5.64 (0.44)	4 mmol BLa^−1^ (W kg^−1^)	4.19 (0.41)	4.12 (0.50)	WT (W kg^−1^)	10.70 (0.70)	10.50 (0.90)	WT (W kg^−1^)	22.90 (2.40)	22.60 (1.70)
Vikmoen, O. et al. (2016)	H	11 (F)	53.50 (3.60)	52.50 (4.20)	W kg^−1^	4.00 (0.30)	4.20 (0.30)	3.5 mmol BLa^−1^ (W kg^−1^)	2.52 (0.35)	2.70 (0.39)	WT (W kg^−1^)	8.10 (0.70)	8.40 (0.60)	WT (W kg^−1^)	17.00 (2.00)	19.10 (2.50)
	C	8 (F)	54.60 (3.40)	53.50 (1.80)	W kg^−1^	4.00 (0.40)	4.20 (0.20)	3.5 mmol BLa^−1^ (W kg^−1^)	2.65 (0.17)	2.76 (0.23)	WT (W kg^−1^)	8.10 (0.50)	8.10 (0.60)	WT (W kg^−1^)	17.70 (1.40)	18.70 (1.70)
Beattie, K. et al. (2017)	H + SL + PL	6 (M)	63.20 (3.16)	59.10 (2.78)	W	391.70 (34.16)	425.00 (41.80)	4 mmol BLa^−1^ (W kg^−1^)	3.55 (0.39)	3.55 (0.45)				Sprint 6 s (W)	864.80 (143.60)	937.70 (116.80)
	C	9 (M)	61.50 (2.58)	59.07 (2.27)	W	408.30 (30.60)	411.10 (30.90)	4 mmol BLa^−1^ (W kg^−1^)	3.74 (0.95)	3.83 (0.79)				Sprint 6 s (W)	925.80 (118.20)	897.20 (124.50)
Del Vecchio, et al. (2019)	H + PL + SL	9 (M)			W	341.00 (62.60)	338.80 (60.00)				Sprint 30 s (W)	8.24 (1.17)	8.50 (1.19)	Sprint 10 s (W kg^−1^)	11.30 (1.80)	11.50 (1.90)
	C	7 (M)			W	362.50 (37.70)	378.10 (48.90)				Sprint 30 s (W)	8.53 (0.95)	8.75 (0.64)	Sprint 10 s (W kg^−1^)	11.60 (1.20)	12.00 (1.10)
Ji, S. et al. (2022)	H	7 (M = 6; F = 1)	55.00 (8.00)	55.00 (10.40)	W	365.00 (71.00)	381.00 (74.00)	4 mmol BLa^−1^ (W)	281.00 (50.00)	286.00 (61.00)	Sprint 15 s (W kg^−1^)	13.65 (2.54)	14.08 (2.65)	Sprint 15 s (W kg^−1^)	10.85 (1.88)	11.32 (2.26)
	H	7 (M = 6; F = 1)	58.30 (8.60)	56.40 (9.10)	W	367.00 (52.00)	391.00 (60.00)	4 mmol BLa^−1^ (W)	280.00 (47.00)	281.00 (52.00)	Sprint 15 s (W kg^−1^)	15.23 (2.81)	15.54 (2.73)	Sprint 15 s (W kg^−1^)	12.50 (2.32)	12.68 (2.32)
	C	6 (M = 5; F = 1)	59.90 (2.70)	58.60 (7.10)	W	336.00 (48.00)	350.00 (44.00)	4 mmol BLa^−1^ (W)	249.00 (30.00)	255.00 (33.00)	Sprint 15 s (W kg^−1^)	14.92 (2.96)	14.04 (3.00)	Sprint 15 s (W kg^−1^)	12.09 (1.74)	11.65 (1.94)
Sitko et al. (2024)	H	12 (M)												Sprint 5 s (W kg^−1^)	15.12 (0.17)	16.31 (0.73)
	C	12 (M)												Sprint 5 s (W kg^−1^)	14.97 (0.22)	14.82 (0.63)

*BLa* blood lactate; *C* control; *F* female; *G* group; *H* heavy strength training; *LT* lactate threshold; *M* male; *n* sample size; *PL* plyometric training; *pVO*_*2*_*max* power related to VO_2_max; *SD* standard deviation; *sec* seconds; *SL* submaximal load strength training; *VT*_*2*_ second ventilatory threshold; *VO*_*2*_*max* maximal oxygen uptake, *WT* Wingate test

**Table 4:** Analysis of cycling efficiency and cycling performance of the included studies

Study	Participant characteristics		Cycling efficiency				Cycling performance		
	G	n	Intensity	Unit of measurement	Mean pre (SD)	Mean post (SD)	Test	Mean pre (SD)	Mean post (SD)
Bishop, D. et al. (1999)	H	14 (F)					60 min all out (W)	186.10 (20.30)	187.90 (20.40)
	C	7 (F)					60 min all out (W)	186.80 (14.50)	192.10 (14.70)
Jackson, N. et al. (2007)	H	9 (M = 8; F = 1)	CE at 150 W	mL kg^−1^ min^−1^	26.70 (3.30)	28.50 (3.40)			
	H		CE at 200 W	mL kg^−1^ min^−1^	33.80 (4.10)	34.90 (3.40)			
	C	5 (M = 3; F = 2)	CE at 150 W	mL kg^−1^ min^−1^	29.50 (4.70)	32.00 (4.80)			
	C		VO_2_ at 200 W	mL kg^−1^ min^−1^	39.30 (4.60)	41.80 (5.30)			
Hausswirth, C. et al. (2010)	H	7 (M)	GE at ~ 240 W	%	18.90 (1.40)	19.00 (1.00)			
	C	7 (M)	GE at ~ 240 W	%	18.20 (1.50)	18.50 (1.20)			
Rønnestad, B. et al. (2010a)	H	6 (M)	CE at 125 W	mL kg^−1^ min^−1^	26.33 (2.89)	25.06 (2.07)	40 min all out (W)	290.50 (31.52)	329.45 (32.08)
	H		CE at 175 W	mL kg^−1^ min^−1^	33.00 (3.51)	33.16 (2.48)			
	H		CE at 225 W	mL kg^−1^ min^−1^	40.42 (4.34)	40.59 (2.07)			
	H		CE at 275 W	mL kg^−1^ min^−1^	48.44 (5.37)	49.11 (1.86)			
	C	6 (M = 5; F = 1)	CE at 125 W	mL kg^−1^ min^−1^	27.04 (2.81)	28.95 (3.37)	40 min all out (W)	288.00 (59.49)	299.04 (67.64)
	C		CE at 175 W	mL kg^−1^ min^−1^	34.30 (3.75)	36.52 (5.81)			
	C		CE at 225 W	mL kg^−1^ min^−1^	41.87 (4.68)	44.47 (5.81)			
	C		CE at 275 W	mL kg^−1^ min^−1^	50.59 (7.49)	53.42 (8.43)			
Sunde, A. et al. (2010)	H	8 (M = 7; F = 1)	GE at 70% VO_2_max (~ 216 W)	%	21.10 (0.70)	22.10 (1.20)	Time until exhaustion at VO_2_max (s)	360.00 (101.00)	422.00 (115.00)
	C	5 (M = 3; F = 2)	GE at 70% VO_2_max (~ 216 W)	%	21.50 (0.90)	21.80 (0.70)	Time until exhaustion at VO_2_max (s)	567.00 (214.00)	597.00 (244.00)
Aagaard, P. et al. (2011)	H	7 (M)	CE at 75% VO_2_max	mL J^−1^	0.20 (0.03)	0.20 (0.01)	45 min time-trial (W)	313.70 (45.90)	340.10 (33.10)
	C	7 (M)	CE at 75% VO_2_max	mL J^−1^	0.22 (0.02)	0.21 (0.01)	45 min time-trial (W)	309.50 (20.30)	321.00 (19.50)
Rønnestad, B. et al. (2011)	H	11(M)					5 min all out trial after 185 min of prolonged cycling (W)	372.02 (28.37)	400.17 (43.01)
	C	9 (M = 7; F = 2)					5 min all out trial after 185 min of prolonged cycling (W)	384.44 (67.88)	380.30 (69.54)
Rønnestad, B. et al. (2015)	H	9 (M)					40 min all out (W kg^−1^)	4.20 (0.52)	4.46 (0.42)
	C	7 (M)					40 min all out (W kg^−1^)	4.48 (0.35)	4.50 (0.48)
Rønnestad, B. et al. (2016)	H	9 (M)	GE at 175 W	%	18.34 (1.05)	18.17 (1.03)			
	C	7 (M)	GE at 175 W	%	18.67 (1.29)	17.89 (1.40)			
Vikmoen, O. et al. (2016)	H	11 (F)	CE at 150 W	mL min^−1^ kg^−1^	38.07 (3.52)	36.71 (3.12)	45 min all out (W kg^−1^)	2.61 (0.24)	2.77 (0.26)
	C	8 (F)	CE at 150 W	mL min^−1^ kg^−1^	35.87 (3.74)	36.18 (4.30)	45 min all out (W kg^−1^)	2.68 (0.22)	2.73 (0.16)
Beattie, K. et al. (2017)	H + SL + PL	6 (M)	CE	mL min^−1^ W^−1^	14.18 (0.89)	14.01 (0.70)			
	C	9 (M)	CE	mL min^−1^ W^−1^	13.72 (0.57)	13.96 (0.36)			
Luckin-Baldwin, K. et al. (2021)	H + SL	15 (M = 10; F = 5)	CE at different power across incremental test	W∙L min^−1^ kg^−1^	67.50 (5.43)	72.58 (5.05)			
	C	15 (M = 12; F = 3)	CE at different power across incremental test	W∙L min^−1^ kg^−1^	68.38 (5.34)	71.70 (4.64)			
Ji, S. et al. (2022)	H	7 (M = 6; F = 1)	CE at 160 W	mL min^−1^ kg^−1^	28.20 (4.70)	28.50 (5.40)	Time until exhaustion at 105% of 4 mmol BLa^−1^ (min)	24.60 (10.10)	35.30 (10.3)
	H		CE at 180 W	mL min^−1^ kg^−1^	31.37 (5.00)	31.17 (5.42)			
	H	7 (M = 6; F = 1)	CE at 160 W	mL min^−1^ kg^−1^	29.50 (2.10)	27.60 (1.70)	Time until exhaustion at 105% of 4 mmol BLa^−1^ (min)	21.10 (8.60)	30.60 (15.70)
	H		CE at 180 W	mL min^−1^ kg^−1^	32.27 (2.51)	30.23 (1.72)			
	C	6 (M = 5; F = 1)	CE at 160 W	mL min^−1^ kg^−1^	32.30 (3.80)	32.80 (5.00)	Time until exhaustion at 105% of 4 mmol BLa^−1^ (min)	17.00 (10.50)	20.70 (6.70)
	C		CE at 180 W	mL min^−1^ kg^−1^	35.42 (4.53)	36.63 (4.97)			
Sitko et al. (2024)	H	12 (M)					20 min all out (W kg^−1^)	4.99 (0.06)	5.21 (0.14)
	C	12 (M)					20 min all out (W kg^−1^)	5.00 (0.08)	5.08 (0.14)

*BLa* blood lactate; *C* control; *CE* cycling efficiency; *F* female; *G* group; *GE* gross efficiency; *H* heavy strength training; *M* male; *n* sample size; *PL* plyometric training; *SD* standard deviation; *SL* submaximal load strength training; *VO*_*2*_*max* maximal oxygen uptake

### Risk of bias, publication bias and certainty assessment

A moderate risk of bias was identified in each of the analyses, with a mean score of 5.53 (Table SM3), mainly due to a lack of group randomisation and/or blinded group allocation. The risk of publication bias was not found in any analysis (Fig. SM1). The certainty of evidence was considered low for each of the analyses, mainly due to the presence of a moderate risk of bias and imprecision (Table SM4).

### Main effects and moderator analysis

**Fig. 2:**
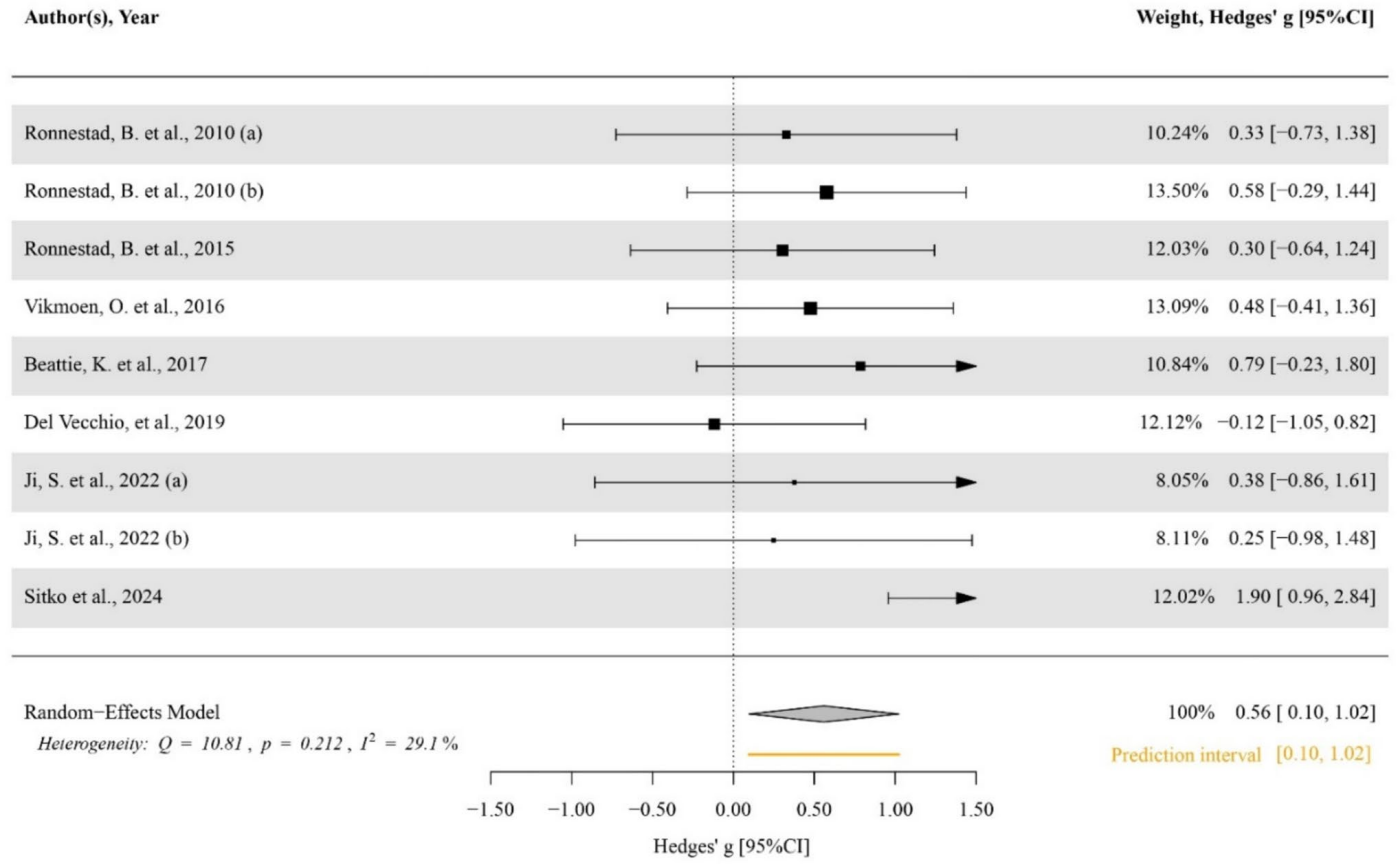
Forest plot of the effect of heavy strength training on anaerobic power. The black squares indicate the mean effect size observed for each study, and their size reflects the study weight. The black lines represent 95% confidence intervals. The diamond represents the pooled effect size (Hedges’ g) with its 95% confidence interval. The orange line represents the prediction interval. A positive effect size represents a beneficial effect, while a negative effect size represents a detrimental effect

**Fig. 3:**
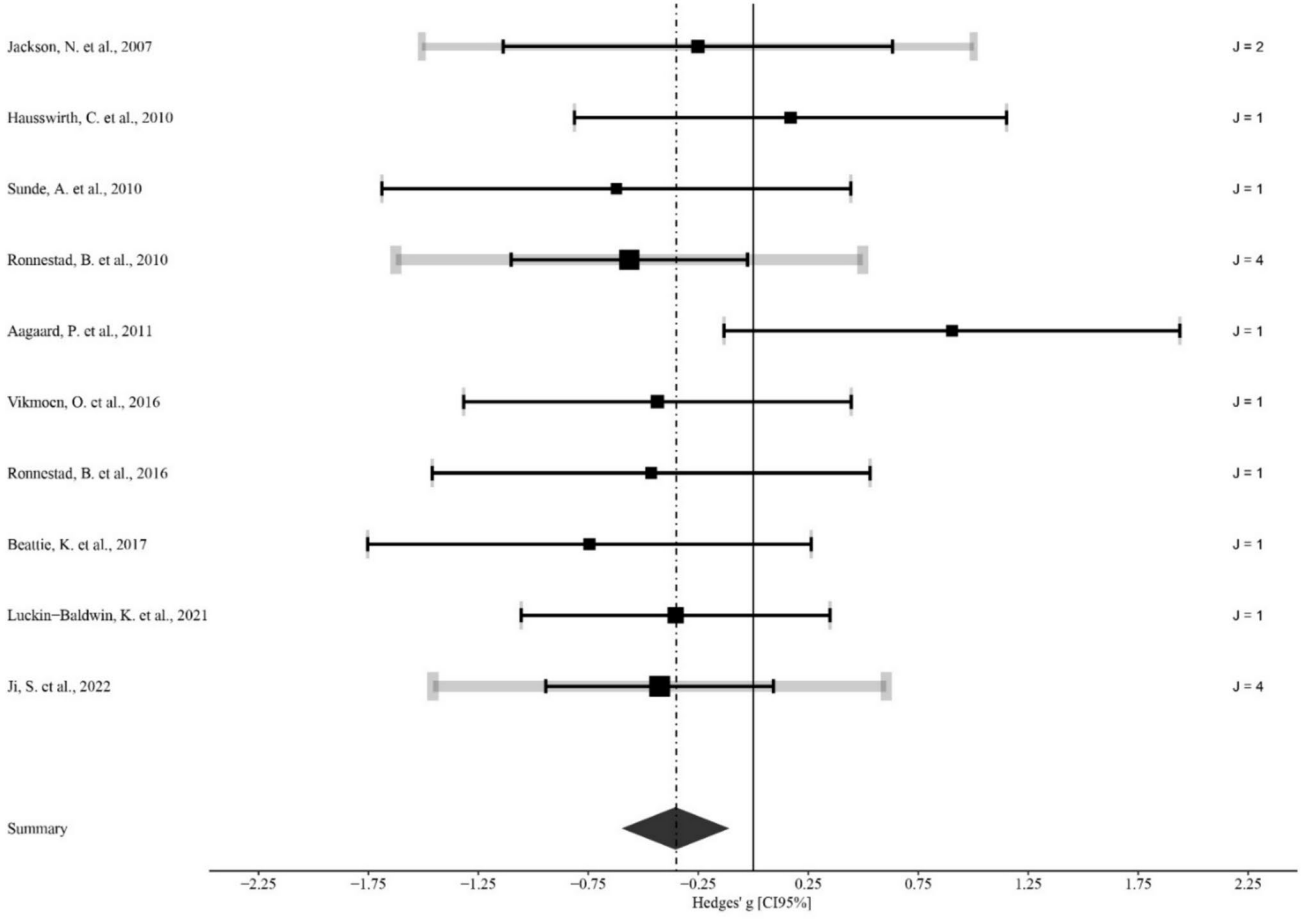
Forest plot of the effect of heavy strength training on cycling efficiency. The black squares indicate the mean effect size observed for each study, and their size reflects the study weight. The black lines represent 95% confidence intervals. The grey line illustrates a 95% confidence interval based on the sampling variance of the individual observed effect sizes, with a thickness proportional to the number of effect sizes reported in each study. The diamond represents the pooled effect size (Hedges' g) with its 95% confidence interval. A negative effect size represents a beneficial effect, while a positive effect size represents a detrimental effect. J, number of effect sizes within studies

**Fig. 4:**
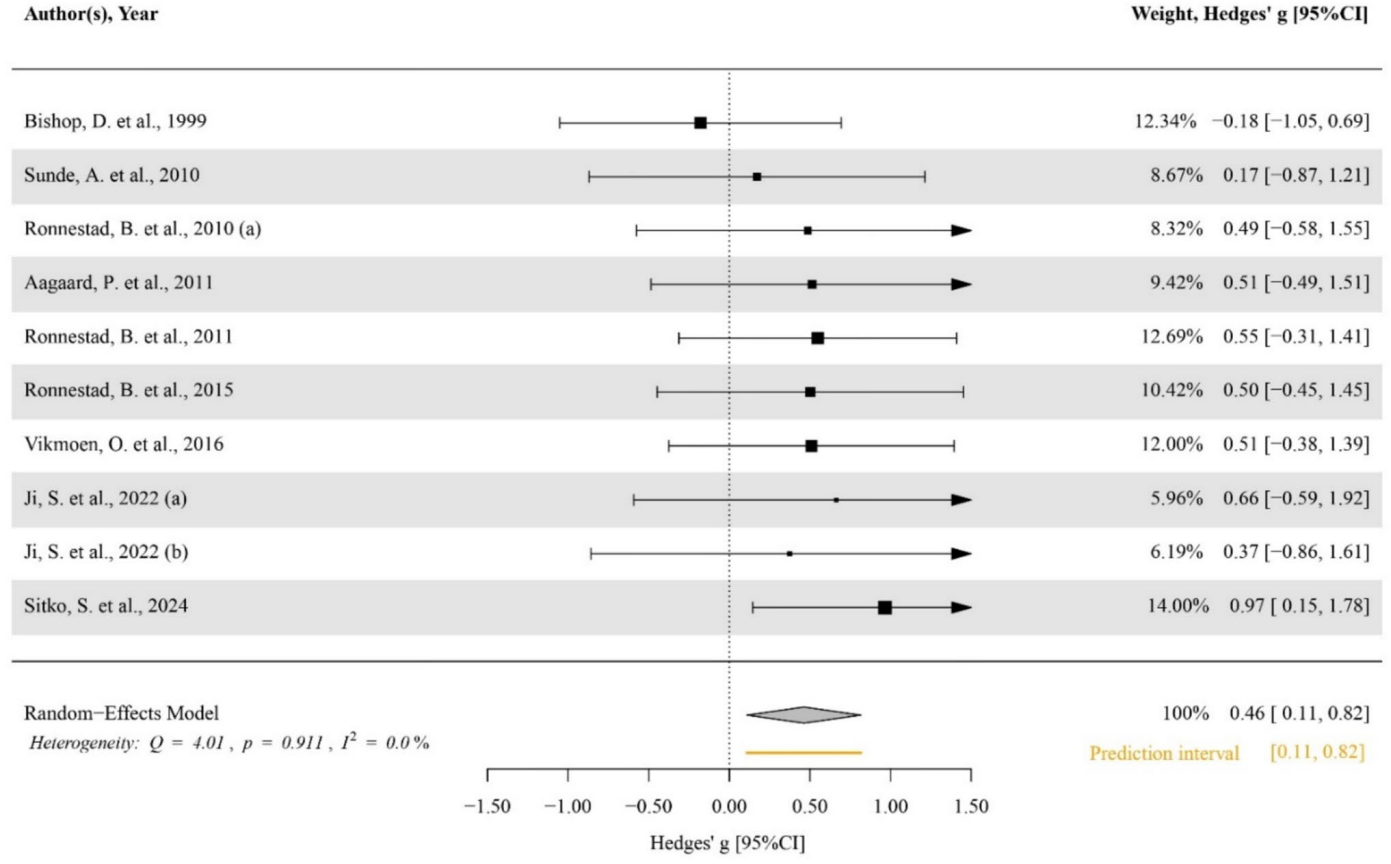
Forest plot of the effect of heavy strength training on cycling performance. The black squares indicate the mean effect size observed for each study, and their size reflects the study weight. The black lines represent 95% confidence intervals. The diamond represents the pooled effect size (Hedges' g) with its 95% confidence interval. The orange line represents the prediction interval. A positive effect size represents a beneficial effect, while a negative effect size represents a detrimental effect

Compared to the control group, there was no significant effect of heavy strength training on VO_2_max (ES [95%CI] = − 0.041 [− 0.345–0.263], *p* = 0.773, *I*^2^ < 0.001, Egger’s test *p* = 0.807; Fig. SM2), pVO_2_max (ES [95%CI] = 0.164 [− 0.176–0.505], *p* = 0.308, *I2* < 0.001, Egger’s test *p* = 0.869; Fig. SM3), MMSS (ES [95%CI] = 0.069 [− 0.323–0.460], *p* = 0.697, *I*^2^ < 0.001, Egger’s test *p* = 0.628; Fig. SM4) and anaerobic capacity (ES [95%CI] = 0.235 [− 0.230–0.700], *p* = 0.263, *I*^2^ < 0.001, Egger’s test *p* = 0.869; Fig. SM5). Whereas compared to control considerations, a significant moderate effect of heavy strength training on anaerobic power was determined (ES [95%CI] = 0.560 [0.097–1.023], *p* = 0.024, *I*^2^ = 29.100, Egger’s test *p* = 0.658; Fig. 2), a small significant effect on cycling efficiency (ES [95%CI] = 0.353 [− 0.619 to − 0.088], *p* = 0.012, *Q*(16) = 10.784, *p* = 0.823, LRT_level2_ = 1, *p* = 1, LRT_level3_ = 1, *p* = 1, Egger’s test *p* = 0.839; Fig. 3) and a moderate significant effect on cycling performance (ES [95%CI] = 0.463 [0.109–0.817], *p* = 0.016, *I*^2^ < 0.001; Egger’s test *p* = 0.844, Fig. 4). Meta-regressions and subgroup analyses showed no significant moderating effect on VO_2_max (all *p* ≥ 0.780, Table SM5) pVO_2_max (all *p* ≥ 0.170, Table SM6), MMSS (all *p* ≥ 0.442, Table SM7), anaerobic capacity (all *p* ≥ 0.567, Table SM8), anaerobic power (all *p* ≥ 0.304, Table SM9), cycling efficiency (all *p* ≥ 0.206, Table SM10), and cycling performance (all *p* ≥ 0.375, Table SM11).

## Discussion

The aim of this systematic review with meta-analysis was to analyse heavy strength training effects on physiological determinants (i.e., VO_2_max, pVO_2_max, MMSS, cycling efficiency, anaerobic capacity, and anaerobic power) of endurance cycling performance (i.e., time to exhaustion and time trial). Heavy strength training produced a small effect on cycling efficiency and a moderate effect on anaerobic power and cycling performance. Heavy strength training may improve cyclist performance, with underlying mechanisms involving improved cycling efficiency and anaerobic power. The results discussed in the following paragraphs are in line with the model proposed by Joyner and Coyle (Joyner and Coyle 2008), who propose that the interaction of VO_2_max, MMSS, non-oxidative energy contribution (i.e., anaerobic power and anaerobic capacity) and cycling efficiency determines endurance performance.

### ***VO***_***2***_***max and MMSS***

Heavy strength training did not improve VO_2_max (ES = − 0.041, *p* = 0.773). The Fick equation indicates that VO_2_max depend on maximal cardiac output (Q_max_) and the arteriovenous oxygen difference (a-vO_2_ difference), thus VO₂max = Q_max_ × a-vO_2_ difference (Skattebo et al. 2020a). The a-vO_2_ difference, dependent on arterial oxygen content (CaO_2_) and mixed venous oxygen content (CvO_2_), is similar in trained and untrained participants (Ekblom et al. 1968), suggesting that training may have a limited impact on VO_2_max through adaptations in the a-vO_2_ difference (Skattebo et al. 2020a). However, improved skeletal muscle fibres capillary and mitochondrial density may increase oxygen extraction (i.e., CvO_2_), thus VO_2_max, and this seems a particularly relevant mechanisms in highly trained individuals (Skattebo et al. 2020a). Skeletal muscle fibres capillary and mitochondrial density improvement is dependent on exercise intensity, requiring mainly efforts that challenge the muscle oxidative capacity (Skattebo et al. 2020b), such as endurance training efforts close to VO_2_max. In contrast, 16 weeks of heavy strength training in highly trained cyclists did not change capillarisation (Aagaard et al. 2011). Moreover, it has been proposed that strength training, when combined with endurance training, may impair mitochondrial remodelling (Zhao and Gao 2024). However, this interference effect appears to be limited in trained individuals and may even be beneficial in untrained individual (Zhao and Gao 2024). Overall, the primary mechanism underlying improvements in VO_2_max after training seems to be the Q_max_ (i.e., product of stroke volume and maximal heart rate) (Montero et al. 2015; Montero and Díaz-Cañestro 2016). Maximal heart rate changes with training does not seem to be related to changes in VO_2_max (Skattebo et al. 2020a), and increases in heart rate may not be energetically favourable for myocardial performance (Heinonen 2025). Therefore, increased stroke volume seems a key mechanism for high VO_2_max (Skattebo et al. 2020a). Stroke volume improvement may be achieved through endurance training efforts requiring (near to) maximal stroke volume (Heinonen 2025), which ranges from ~ 40 to 100% VO_2_max in untrained to trained individuals, respectively (Vella and Robergs 2005). Therefore, heavy strength training may fail to appropriately stimulate the main underlying mechanisms associated with VO_2_max (Heinonen 2025), in line with our meta-analyses.

The MMSS was not affected by heavy strength training (ES = 0.069, *p* = 0.308). This physiological determinant is defined as the highest oxidative metabolic rate that can be maintained during continuous exercise (Jones et al. 2019) and is independently affected by convective oxygen supply, diffusive oxygen transport and oxygen utilisation (Goulding and Marwood 2023). Convective oxygen supply refers to the transport of oxygen through the circulatory system to active muscles (Goulding and Marwood 2023). This process is influenced by the duty cycle of muscle contraction, as blood flow is restricted during contraction and increases during relaxation due to changes in intramuscular pressure and compression of blood vessels (Goulding and Marwood 2023). This mechanism may explain the transient increase in muscle blood flow observed after the pedal thrust phase of the crank cycle, probably because of a brief occlusion during contraction (Takaishi et al. 2002). Supporting this idea, a study found a significant pre-post intervention effect on MMSS (measured as power output at 4 mmol BLa^−1^), which correlated with a shift towards early peak torque during pedal stroke (r = − 0.50, *p* = 0.05) (Rønnestad et al. 2015), suggesting a reduced duration of blood flow occlusion and, consequently, enhanced convective oxygen supply. In contrast, several studies did not report a significant effect of heavy strength training on MMSS (Bishop et al. 1999; Hausswirth et al. 2010; Sunde et al. 2010; Aagaard et al. 2011; Beattie et al. 2017; Ji et al. 2022). This discrepancy may be explained by the other two limiting factors of MMSS (i.e., diffusive oxygen transport and oxygen utilization). Diffusive oxygen transport refers to the movement of oxygen from the capillaries to the muscle mitochondria and is primarily determined by muscle capillarity (Mitchell et al. 2018; Goulding and Marwood 2023), particularly the capillarization of type I muscle fibres (Mitchell et al. 2018). As previously discussed, no significant changes in capillarization have been observed following heavy strength training in highly trained cyclists (Aagaard et al. 2011), which may explain the lack of improvement in this component of oxygen delivery. As for oxygen utilisation, this is determined in part by the oxidative capacity of the muscle (Jones et al. 2019; Goulding and Marwood 2023; Peden et al. 2024), with MMSS showing a strong correlation to mitochondrial content (r = 0.88, *p* < 0.001) (Peden et al. 2024). However, two studies (Bishop et al. 1999; Vikmoen et al. 2016) failed to find significant expression of aerobic enzymes (specifically citrate synthase, hydroxyacyl-CoA dehydrogenase, cytochrome c oxidase subunit IV, and 2-oxoglutarate dehydrogenase). Considering the above, improvements in convective oxygen delivery (potentially due to increased blood flow from reduced muscle contraction time during the pedal thrust phase), appear less critical for enhancing MMSS than improvements in diffusive oxygen transport and, primarily, oxygen utilization capacity. Therefore, it is possible that heavy strength training is of limited relevance for improving MMSS.

On the other hand, “interference effect” of concurrent training, arising from potential conflicts between molecular regulators of muscle metabolism and acute residual fatigue from strength training) may impair aerobic adaptations (Coffey and Hawley 2017). Hickson’s seminal study (Hickson 1980), observed a plateau in strength gains (1RM) without changes in VO_2_max, but a separate study the same year (Hickson et al. 1980), reported a modest VO_2_max increase (~ 4%) and a substantial improvement in time to exhaustion at VO_2_max (~ 47%). These results suggest that while interference may hinder hypertrophy and strength, it does not necessarily impair oxidative metabolism (Coffey and Hawley 2017). This is supported by meta-analyses showing no significant effects of heavy strength training on VO_2_max and MMSS in endurance runners and skiers (Castañeda-Babarro et al. 2022; Llanos-Lagos et al. 2024a)., evidence indicates that heavy strength training does not meaningfully improve VO_2_max or MMSS but also does not negatively affect these factors. Therefore, the exclusion of heavy strength training should not be based solely on concerns about interference with aerobic adaptations.

### ***pVO***_***2***_***max***

The pVO_2_max, a metric that reflects the interaction between VO_2_max, cycling efficiency and anaerobic performance (Jones and Carter 2000), it is considered a good predictor of cycling performance (Balmer et al. 2000; Bentley et al. 2001). In our analysis, we did not observe a significant improvement in this metric (ES = 0.164, *p* = 0.308), despite finding improvements in cycling economy and anaerobic power. It is possible that a significant effect on pVO_2_max was not found because an improvement in cycling efficiency and/or anaerobic power is not a sufficient stimulus to increase this variable, as an increase in VO_2_max would be. On the other hand, the different protocols used could affect the accuracy of the detection of the changes produced by heavy strength training. For example, there were studies that measured pVO_2_max with long (i.e., ≥ 3 min) (Jackson et al. 2007; Levin et al. 2009; Ji et al. 2022) and short (i.e., ≤ 1 min) (Rønnestad et al. 2010a, b, 2015; Hausswirth et al. 2010; Vikmoen et al. 2016; Beattie et al. 2017; Del Vecchio et al. 2019) duration stages. In fact, it is proposed that short-duration stages are more suitable for measuring pVO_2_max (Panissa et al. 2017). However, researchers (Sunde et al. 2010) who also measured time to exhaustion at pVO_2_max intensity found a significant improvement after the inclusion of heavy strength training. It is, therefore, suggested that future research should consider using appropriate protocols to determine the changes in pVO_2_max generated by strength training.

### Cycling efficiency

Our meta-analysis showed that heavy strength training improves cycling efficiency (ES = 0.353, *p* = 0.012). For an explanation of these results, it is necessary to discuss the possible mechanisms by which this training method could improve cycling efficiency. From strength training, we can achieve different neuromuscular adaptations, which we can classify (although not independent of each other) as neurological adaptations (i.e., central adaptations) and morphological adaptations (i.e., peripheral adaptations) (Folland and Williams 2007; Suchomel et al. 2018). Within the neurological adaptations, it is known that heavy strength training can improve motor unit recruitment and firing frequency, which may result in an improvement in maximal muscle strength (e.g., maximal voluntary contraction) and rate of force development (RFD) (Aagaard et al. 2002, 2011). About maximal muscle strength, several studies included in the analysis have reported an improvement in maximal muscle strength in lower limbs, measured as maximal force in isometric half squat (Rønnestad et al. 2010b, 2015), isometric quadriceps (Aagaard et al. 2011), isometric mid-thigh pull (Beattie et al. 2017), isometric leg press, leg extension and leg curl (Ji et al. 2022). Therefore, if this gain in maximal muscle strength (which was measured off the bike) were to be translated on the bike, the force required for each pedal thrust would become a smaller percentage of the new maximal force (Hickson et al. 1988; Aagaard et al. 2011). Along the same lines, in accordance with the principle of motor unit recruitment (Henneman et al. 1965) and considering that slow fibres (i.e., fibres I type) are more efficient than fast fibres (i.e., type II fibres) (Coyle et al. 1992), a lower relative force demand would favour a greater involvement of slower fibres. This would delay the activation of the faster fibres, thus improving cycling efficiency. Regarding RFD, two studies reported an improvement in this measure during squat 90° (Sunde et al. 2010) and isometric quadriceps (Aagaard et al. 2011). This improvement could be related to an earlier peak torque improvement during the propulsive phase of pedalling (Aagaard et al. 2011), as has been found in a later study (Rønnestad et al. 2015). For example, it has been reported that in isometric contractions the energy cost is higher at the beginning of the muscle contraction than during the maintenance phase of contraction (Russ et al. 2002). Therefore, if this metabolic behaviour occurs during muscle contraction in the pedal stroke, an early torque peak would reflect a decrease in force generation time, increasing force maintenance time, decreasing the energy cost of muscle contraction and thus improving cycling efficiency. Moreover, Hansen et al. (2012) (a follow-up to a study in this meta-analysis (Rønnestad et al. 2011)) found that heavy strength training reduced negative crank torque during the upstroke phase. This was linked to increased hip flexor activation, possibly due to improved RFD, tendon stiffness, or muscle activation timing. Similarly, professional cyclists exhibit lower negative crank torque in the upstroke than elite and club-level cyclists (García-López et al. 2016). Enhanced hip flexor activation may lessen extensor muscle workload (Hansen et al. 2012), potentially improving cycling efficiency. However, these studies did not directly assess cycling efficiency or its mechanisms, highlighting the need for further research.

Morphological changes such as a change in fibres IIx to IIa have also been postulated to improve cycling efficiency (Aagaard and Andersen 2010; Rønnestad and Mujika 2014; Mujika et al. 2016). For example, two studies reported an increase in type IIa fibres with a decrease in type IIx fibres after 16 weeks (Aagaard et al. 2011) and 11 weeks (Vikmoen et al. 2016) of heavy strength training in male and female cyclists, respectively. While another study showed no changes in muscle fibres characteristics (i.e., changes in fibre percentage, fibre area and fibre least diameter) (Bishop et al. 1999). The increase in type IIa fibres at the expense of IIx fibres, which are less prone to fatigue and have a higher power output compared to slower fibres (Bottinelli et al. 1999), could improve cycling performance (Aagaard et al. 2011; Vikmoen et al. 2016) and cycling efficiency (Vikmoen et al. 2016). However, these changes have not been accompanied by an improvement in cycling efficiency (Aagaard et al. 2011) or have not correlated with the change in cycling efficiency (Vikmoen et al. 2016). Therefore, further research is required to clarify the effect on fibre type change and its relationship to cycling efficiency. In addition, an increase in the CSA of the quadriceps femoris muscle has been correlated with cycling efficiency (r = 0.535, *p* < 0.002) (Vikmoen et al. 2016). This could lead to an increase in force production, allowing for greater maximal force, and as mentioned above, a decrease in the relative force in the pedal stroke.

Of note, the results presented in this meta-analysis are based on changes in cycling efficiency in the non-fatigued state and at submaximal intensity (i.e., intensity equal to or less than the MMSS). Nevertheless, it is well known that cycling efficiency declines over prolonged efforts (Passfield and Doust 2000; Noordhof et al. 2015; Hopker et al. 2017; Stevenson et al. 2022), which may explain the decrease in performance over prolonged effort (Passfield and Doust 2000). The appearance and magnitude of any degradation of physiological determinants (in this case, cycling efficiency) over time during prolonged exercise may be due to durability (also called resilience) (Maunder et al. 2021; Jones 2023). During efforts in moderate and heavy intensity domains (i.e., at intensities below MMSS), an impairment of contractile function might be observed, which may be mainly due to a depletion of glycogen stores, leading to a reduction of Ca^2+^ release from the sarcoplasmic reticulum, reducing neuromuscular function (Brownstein et al. 2021). Indeed, glycogen depletion increases the recruitment of fast fibres and increases oxygen demand (Krustrup et al. 2004). Therefore, it is possible that, at the beginning of a moderate intensity effort, in some cases, there may not be a clear difference in cycling efficiency after a heavy strength training programme, but may be noticeable in a fatigued state. For instance, no improvement in cycling efficiency at the beginning of exercise, but improvement after two hours of exercise at submaximal intensity (i.e., at 44% of pVO_2_max) has been reported in two studies (Rønnestad et al. 2011; Vikmoen et al. 2017). Although in another study (Hausswirth et al. 2010), no improvement in cycling efficiency was noted at any point during prolonged effort (i.e., at first ventilatory threshold + 3% [~ 73% VO_2_max]), a maintenance of pedalling frequency and a stabilisation of the electromyographic activity of the vastus lateralis were observed. This suggests that recruitment of the new faster fibres was not necessary, which may result in a delay in the impairment of cycling efficiency (i.e., improved durability). Along the same lines, Rønnestad et al. (2010a) reported that after 25 weeks of heavy strength training, the cycling efficiency in non-fatigued state was also not improved, but the respiratory exchange rate was observed to decrease from baseline in the strength training group during a continuous incremental test, suggesting an increased ability to utilize fatty acids as an energy source, or a recruitment mainly of type I fibres. Therefore, it is arguable that the accumulation of neurological and morphological adaptations given by heavy strength training, such as increased maximal strength and improved RFD during pedalling, as well as an increase in type IIa fibres at the expense of type IIx fibres, may lead to a greater predominance of type I fibre recruitment, delaying the recruitment of faster and possibly less economical fibres (Coyle et al. 1992), improving or maintaining cycling efficiency during prolonged efforts at submaximal intensities, which could impact on durability.

### Non-oxidative energy contribution

Non-oxidative energy contribution was analysed as anaerobic capacity and anaerobic power. Heavy strength training had no effect on anaerobic capacity (ES = 0.235, *p* = 0.263) and a significant effect on anaerobic power (ES = 0.560, *p* = 0.024). Regarding anaerobic capacity, given that glycolytic metabolism is predominant (Spriet et al. 1989), it is to be expected that heavy strength training is not enough stimulus to generate metabolic changes. However, heavy strength training could eventually influence anaerobic power. From the studies included in this meta-analysis, three studies reported that heavy strength training had a significant effect compared to the control group on peak power output in the Wingate test (Rønnestad et al. 2010a, b) and the 6-s sprint (Beattie et al. 2017), while other studies reported a significant effect pre–post training (Vikmoen et al. 2016), and a tendency to improve (Rønnestad et al. 2015) in the Wingate test. Anaerobic power (i.e. peak power output) depends on the interaction between force production and muscle contraction velocity, and the latter depends on the size of the muscle and the proportion of fast-twitch fibres recruited (Sargeant 2007; Galvan-Alvarez et al. 2024). Of note, quadriceps morphology (e.g., increased muscle size) is one of the mechanisms by which strength training can increase peak power output (Kordi et al. 2020; Galvan-Alvarez et al. 2024). For instance, two studies that improved anaerobic power (i.e., peak power output) also improved thigh muscle CSA while performing two heavy strength training sessions for twelve weeks (Rønnestad et al. 2010a, b) and eleven weeks (Vikmoen et al. 2016). While the other studies reported a significant increase in lean leg mass (Rønnestad et al. 2015; Beattie et al. 2017). In fact, a correlation has been found between increased anaerobic power and increased CSA of the knee extensors (r = 0.47, *p* < 0.05) (Rønnestad et al. 2010b). On the other hand, two studies failed to find a significant improvement in anaerobic power in a 10-s sprint in masters cyclists (Del Vecchio et al. 2019) and in a 15-s sprint in groups of cyclists who performed bilateral or unilateral exercises (Ji et al. 2022). Possibly, these studies failed to find an improvement due to the protocol used to analyse anaerobic power. For example, the peak power output is reached during the first 5 s of the effort and then decreases by 20–50% in a 15-s effort (Williams et al. 1988). Due to the longer sprint protocols (i.e., 10- and 15-s), it is possible that the cyclists may have dosed the effort, avoiding the elucidation of these possible changes. In addition, in the study of master cyclists (Del Vecchio et al. 2019), no significant changes in whole body lean mass were found compared to the control group, possibly due to factors related to age-related decline in muscle strength (Del Vecchio et al. 2019). Increased anaerobic power has also been related to neurological factors, such as improved maximal muscle strength and RFD (Stone et al. 2004; Douglas et al. 2021), as found in several studies that showed an improvement in maximal muscle strength (Rønnestad et al. 2010b, 2015; Beattie et al. 2017; Ji et al. 2022) and possibly an improvement in RFD (i.e., increased early peak torque) (Rønnestad et al. 2015). In fact, an improvement in anaerobic power has been found in values relative to body weight (i.e., peak power output [W kg^−1^]) (Rønnestad et al. 2010b) thus it is possible to assume that this improvement could be due to neurological improvements. Moreover, intermuscular coordination, which refers to the efficiency with which specific muscle recruitment patterns occur to optimise performance of a given task (Carroll et al. 2001), is key when considering transfer from off-bike strength exercises to cycling. Off-bike exercises like squats or deadlifts involve different coordination patterns than pedalling, which may limit transfer of strength gains to cycling (Koninckx et al. 2010). However, studies with track sprint (Burnie et al. 2022) and endurance cyclists (Koninckx et al. 2010) show these strength gains can increase peak power output, suggesting intermuscular coordination may adapt specifically to cycling demands despite differing initial movement patterns. Yet, single-joint or machine-based exercises may lack this specificity (Carroll et al. 2001; Worn et al. 2024). For example, Ji et al. (2022) used only machine-based exercises, while Del Vecchio et al. (2019) regarding heavy strength training exercises, only single-leg leg press and hip flexion were included. In contrast, the other studies (Rønnestad et al. 2010a, b, 2015; Vikmoen et al. 2016; Sitko et al. 2024) used a broader range of free-weight, single- and multi-joint exercises. Thus, exercise selection likely affects transfer to cycling, but further research is needed (Carroll et al. 2001).

Taken together, it is expected that heavy strength training may improve anaerobic power without changes in anaerobic capacity. These improvements may be especially relevant in competitions or race segments where the intensity of MMSS is exceeded. For example, in road cycling competitions, although a large part of the time is spent pedalling at low intensities, non-oxidative energy demand can suddenly increase (i.e., exceeding the MMSS intensity) (Erp et al. 2021; Areta et al. 2024), such as during tactical positioning in the peloton, overtaking other cyclists, or a sprint finish (Faria et al. 2005). Similarly, in Olympic cross-country mountain biking, the time spent above the MMSS intensity exceeds 40.0% of the race (Granier et al. 2018). Indeed, anaerobic power and Olympic cross-country performance have been correlated (r = 0.38, *p* < 0.05) (Hays et al. 2021). Therefore, improving anaerobic power may be relevant in cyclists whose role in competition and/or the competition itself requires higher levels of peak power output.

### Cycling performance

Heavy strength training was reported to have a moderate effect on performance (i.e., in time trials and time to exhaustion) in endurance-trained cyclists (ES = 0.463, *p* = 0.016). Following a framework that divides the determinants of performance in endurance sports into metabolic (i.e., VO_2_max, MMSS, cycling efficiency and anaerobic capacity) and non-metabolic (i.e., cycling efficiency and anaerobic power) factors (Paavolainen et al. 1999; Hayes and Gordon 2021), we might assume that heavy strength training improves cycling efficiency and anaerobic power through non-metabolic adaptations, which are reflected in improved cycling performance. In fact, several correlations have been found between the change after including heavy strength training in variables related to cycling efficiency, anaerobic power and cycling performance that could support these results. For example, a significative correlation has been found between the change in cycling efficiency and cycling performance (i.e., mean power output during 40 min time trial and time to exhaustion at pVO_2_max) after the addition of heavy strength training (Sunde et al. 2010; Vikmoen et al. 2016), whereas a significant correlation between the change in cycling performance (i.e., mean power output during 40 min time trial) and change in CSA (Vikmoen et al. 2016) and a tendency correlation with pVO_2_max (*p* = 0.06) (Rønnestad et al. 2015).

The included studies measured cycling performance at heavy (i.e., below MMSS) (Bishop et al. 1999; Rønnestad et al. 2010a, 2015; Aagaard et al. 2011; Vikmoen et al. 2016) and severe domain (Sunde et al. 2010; Ji et al. 2022; Sitko et al. 2024) intensities (i.e., above MMSS). Considering that cycling efficiency was measured at heavy and moderate domain intensities, it is possible that these improvements will also be reflected in severe domain intensities. At intensities in the severe domain, as opposed to the heavy domain, the duration of effort is limited first by alterations of the metabolic milieu (related to peripheral fatigue (Blain et al. 2016)) and preceded by impairment of contractile function (Brownstein et al. 2021). Given that no improvements in MMSS was found, which could establish an improved upper limit preventing or delaying the transition to an altered metabolic state, nor an improvement in anaerobic capacity that would allow a longer duration of effort from the non-oxidative energy contribution, it is possible that central neural enhancement, such as improved motor unit recruitment and firing rate modulation (resulting from heavy strength training) has a compensatory effect on peripheral fatigue (Black et al. 2017). In fact, athletes with less force reduction at this intensity may be able to reduce the increase in energy cost (Hayes et al. 2011), resulting in an improvement in the duration of time until exhaustion. On the other hand, performance in a fatigued state was also included (Rønnestad et al. 2011). In this study it was found that cyclists improved their 5 min all-out effort after 185 min at 44% of pVO_2_max, whereas in a similar study (Vikmoen et al. 2017) in female cyclists (results not included in the analysis) an improvement in the similar cycling test (i.e., 5 min all-out effort after 180 min at 44% of pVO_2_max) was also found. These results could be due to improved cycling efficiency in the fatigued state, as discussed above.

### Characteristics of participants and strength training intervention

A series of subgroup and meta-regression analyses were conducted to identify possible moderating factors. Although we found no moderating effect of participants characteristics and strength training intervention on the variables analysed, it is important to mention some points. No moderating effect of sex on performance (or its determinants) was observed (all *p* > 0.170), in line with a review that noted similar strength training benefits in male and female cyclists (Vikmoen and Rønnestad 2021). Moreover, one study (Vikmoen et al. 2016) reported that females increased cycling performance, anaerobic power, cycling efficiency, muscle CSA, and skeletal muscle fibre shift from type IIa to type IIx, resembling our results from studies including males. Nonetheless, only two studies in our systematic review recruited female-only participants (Bishop et al. 1999; Vikmoen et al. 2016). Therefore, more research is needed before a robust interpretation on the role of biological sex as a moderating factor for the effect of strength training on cycling performance. Aside from participants sex, the strength training effect on cycling performance (or its determinants) may be moderated according to participants level before intervention (e.g., reduced adaptive response in highly trained athletes) (Aagaard and Andersen 2010). However, our analyses indicated that cycling performance changes after strength training were independent from athletes initial VO_2_max values (i.e., from 48.3 to 75.5 mL kg^−1^ min^−1^). Nonetheless, a moderator role for athlete’s initial fitness level cannot be ruled out due to the very low certainty of the evidence.

Regarding characteristics of the intervention, it was reported that training programmes lasted between 5 and 25 weeks, with a training frequency of 1–3 sessions per week. Among the studies with shorter durations of intervention, the study by Sunde et al. (2010) was the first to report an improvement in cycling efficiency and performance (i.e., time to exhaustion at pVO_2_max) after 8 weeks of training. While another study (Hausswirth et al. 2010) of 5 weeks duration did not report an improvement in cycling efficiency. At the other end, it was reported that 25 weeks of training starting with a 12-week preparatory period with 2 weekly strength training sessions improved muscle strength and muscle CSA (Rønnestad et al. 2010a). Then, in a 13-week competitive period, one session per week was sufficient to sustain these improvements. This approach resulted in improved cycling performance compared to the control group. Additionally, adaptations obtained by strength training may be maintained up to 8 weeks after training cessation (Rønnestad et al. 2016), or up to 6 weeks (Bláfoss et al. 2022). Therefore, effective strength training interventions may require 8 weeks and/or 2 training sessions per week, and its effects might last for up to 6–8 weeks after cessation of this type of training. Several studies (Jackson et al. 2007; Levin et al. 2009; Rønnestad et al. 2015; Vikmoen et al. 2016; Del Vecchio et al. 2019) reported > 80.0% of adherence to strength training sessions. However, one study (Jackson et al. 2007) reported that athletes considered the additional strength training sessions as unsustainable over time. Moreover, one study (Sunde et al. 2010) reported a 12.2% decrease in cycling training time. Indeed, all but two studies (Rønnestad et al. 2015; Del Vecchio et al. 2019) added strength training sessions to endurance training. Therefore, to increase adherence, strength training load probably should be adapted (e.g., replace a portion of other training components) according to athletes’ past-current cycling training schedule, considering key prescription factors (e.g., frequency, season period, inter-individual responses).

The limitations and strengths of this systematic review with meta-analysis are important to note. First, the limited number of participants included (i.e., imprecision) and the moderate risk of bias (mainly absence of random and concealed allocation of groups, Table SM3) in all analyses are the main reasons for the low certainty of the evidence (Table SM4). Second, all studies reported that subjects either had no previous systematic strength training experience or it was not reported, and the longest intervention duration was 25 weeks thus the results could be blunted in athletes undergoing a longer period of systematic strength training (Van Hooren et al. 2024). Third, all but two studies (Rønnestad et al. 2015; Del Vecchio et al. 2019) reported that strength training replaced part of the endurance training to maintain the same volume of hours. Therefore, coaches and practitioners should consider several factors when deciding between adding or substituting strength training for endurance training, considering such factors as athlete availability, programme planning and control of training loads. Fourth, the underrepresentation of female participants relative to males may reduce statistical power in the subgroup analysis, leading to a Type II error (i.e., false negative). Fifth, although all control and strength training groups performed endurance training, variations in the structure and content of endurance training schedules may have influenced the adaptations attributed to strength training (Berryman et al. 2019). Sixth, the protocol was registered after the data analysis (Afonso et al. 2024). However, the protocol followed previous systematic review guidelines (Llanos-Lagos et al. 2024a, b), and the current analyses included several improvements. Indeed, the strengths of this meta-analysis should also be acknowledged. To our knowledge, this is the first meta-analysis in endurance cyclists to examine the effect of heavy strength training on physiological determinants of cycling performance. Although the analyses are not sufficient to determine the mechanisms underlying an improvement in cycling performance, it allows us to establish pathways for future original studies.

## Practical applications

**Fig. 5:**
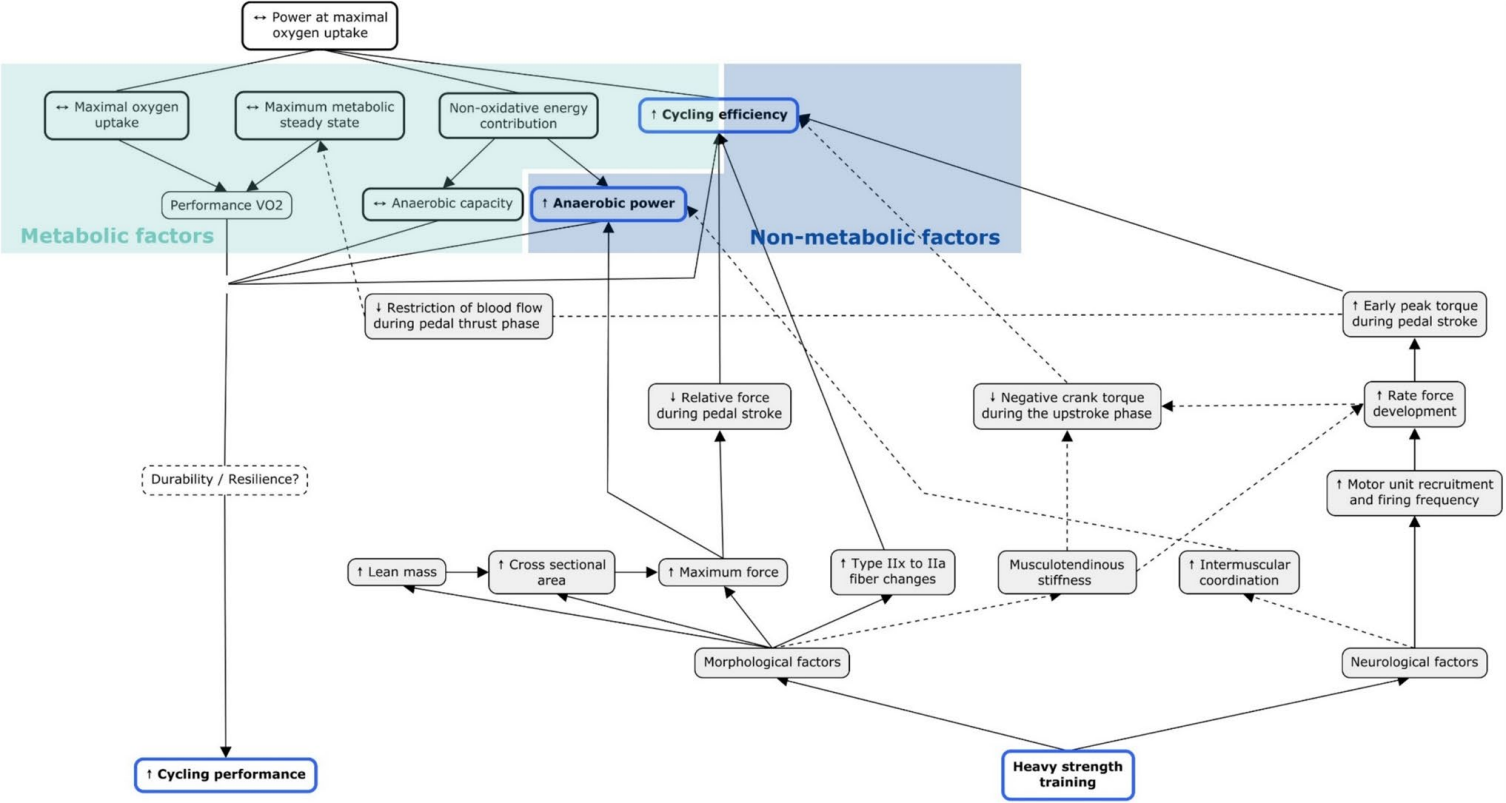
Proposal of possible mechanisms by which heavy strength training might improve physiological determinants and cycling performance. The physiological determinants of endurance cycling performance is based on the model of Joyner and Coyle (Joyner and Coyle 2008), who propose that the interaction of maximal oxygen uptake, maximum metabolic steady state, non-oxidative energy contribution (i.e., anaerobic power and anaerobic capacity) and cycling efficiency determine cycling performance, and updated by Jones (Jones 2023), which includes resilience/durability. In addition, these physiological factors are categorised as metabolic factors (highlighted in the light blue pentagon) and non-metabolic factors (highlighted in the blue) (Paavolainen et al. 1999; Hayes and Gordon 2021). Blue rounded rectangles represent a significant effect (*p* ≤ 0.05) from meta-analysis results. Rounded grey rectangles and grey lines show the mechanisms and their pathways by which heavy strength training could improve physiological determinants of performance. Solid lines indicate well-explored mechanisms, while dashed lines represent less explored mechanisms that may also play a role. Durability is represented as a rounded rectangle with dashed lines because this variable was not analysed in this study. VO_2_, oxygen uptake; ↑ denotes an increase/improvement; ↓ denotes a decrease; ↔ denotes unchanged

The inclusion of heavy strength training (i.e., ≥ 80% 1RM) in the training programme for cyclists is an effective strategy to improve cycling performance, measured as time to exhaustion and time to time trial. The characteristics of the strength training programmes included in the analysis and the practical recommendations for coaches and practitioners are summarised in Table 5. Although this study does not directly analyse the factors that explain the improvement in performance, from the model of physiological determinants of endurance performance, it can be assumed that this progress may be linked to an improvement in cycling efficiency and anaerobic power. Importantly, heavy strength training does not affect VO_2_max, pVO_2_max, MMSS or anaerobic capacity. A proposed model related to these results is presented (Fig. 5). Therefore, coaches and practitioners should consider integrating heavy strength training into their training programmes, especially if they are looking to improve performance through improved cycling efficiency and anaerobic power.

**Table 5:** Summary of the characteristics of the strength training programmes included in the analysis and practical recommendations

Variable	Exercises*	Training load*		Programme duration	
	Mean (min–max)	Variable	Mean (min–max)	Variable	Mean (min–max)
Nº of exercises per session	~ 4 (1–7)	Load (%1RM; RM)	~ 84% 1RM or ~ 7RM (67% 1RM to 95 or 12RM to 2RM)	Duration (weeks)	~ 14 (5–25)
Bilateral exercises (study frequency)	Squat (12), ankle plantar flexion (6), leg curls (5), leg press (4), leg extension (3), calf raises (2), hip flexion (1), toe raise (1), deadlift (1), glute hamstring raises (1), hip thrust hip abduction (1)	Sets	~ 3.5 (1–5)	Sessions per week	~ 2 (1–3)
Unilateral exercises (study frequency)	Single-leg leg press (10), single-leg hip flexion (8), single-leg leg curls (1), single-leg step up (1), lunges (1), straight-leg deadlift (1), single-leg calf raises (1), single-leg leg extension (1)	Rest (min)	2.5 (1–3)	Total sessions	~ 28 (15–48)
Recommendation	4 exercises (2 bilateral and 2 unilateral exercises). For example, squat, single-leg press, hip flexion, and single-leg calf raises	Progressive loads from 70 to 90% 1RM or 11RM to 4RM, with 3–4 sets per exercise, with 2–3 min rest		Minimum 8 weeks duration with 2 sessions per week (1 session/week to maintain improvements)	

*Max* maximum, *min* minimum; *RM* repetition maximums; *1RM* one repetitions maximum

*The mean values (minimum and maximum) are calculated exclusively from heavy strength training of the studies included

## Conclusion

Heavy strength training can improve endurance cycling performance (i.e., time to exhaustion and time trial), in line with improved cycling efficiency and anaerobic power, without changes in VO_2_max, pVO_2_max, MMSS, and anaerobic capacity. Improvements seem likely after 8 weeks, and/or with 2 sessions per week, including 4 lower-body exercises were included, comprising both bilateral and unilateral movements, performed for 3–4 sets per exercise. However, the low certainty of the evidence precludes a robust recommendation regarding optimal heavy strength training prescription. For more robust recommendations, future research is advised to incorporate a larger sample size (especially in females’ athletes) and randomly assign and conceal the groups. Additionally, research exploring the mechanisms underlying the effects of heavy strength training is advised, and to compare with other strength training methods (e.g., plyometric training, strength training with submaximal loads) and training methodologies (e.g., velocity-based training).

## Supplementary Information

Below is the link to the electronic supplementary material.

Supplementary file1 (DOCX 1381 KB)

## Data Availability

All data generated or analysed during this systematic review and meta-analysis are included in the article as table(s), figure(s), and/or Online Supplementary Material(s). Any other data requirement can be directed to the corresponding author upon reasonable request.
