# Supplementary material for: Heavy strength training effects on physiological determinants of endurance cyclist performance: a systematic review with meta-analysis
Source: Eur J Appl Physiol. 2025 Jul 9;126(1):193–222. doi: 10.1007/s00421-025-05883-2 (PMC12881108; doi:10.1007/s00421-025-05883-2)
Supplement: Supplementary file 1 — Supplementary file1 (DOCX 1381 KB) [file 421_2025_5883_MOESM1_ESM.docx]

**Supplementary material**

**Heavy strength training effects on physiological determinants of endurance cyclist performance: a systematic review with meta-analysis**

Cristian Llanos-Lagos (ORCID: 0009-0003-6906-4977) ^1^, Rodrigo Ramirez-Campillo (ORCID: 0000-0003-2035-3279) ^2,3,4^, Eduardo Sáez de Villarreal (ORCID: 0000-0001-9583-9287) ^1^

^1^ Physical Performance Sports Research Centre (PPSRC), Universidad Pablo de Olavide, 41704 Sevilla, Spain.

^2^ Exercise and Rehabilitation Sciences Institute. School of Physical Therapy. Faculty of Rehabilitation Sciences. Universidad Andres Bello. Santiago 7591538, Chile.

^3^ Laboratorios de Ciencias del Deporte y Rendimiento Humano, Instituto de Alta Investigación, Universidad de Tarapacá, Casilla 7D, Arica, Chile.

^4^ Department of Physical Activity Sciences. Universidad de Los Lagos. Chile.

**Corresponding author**

Prof. Eduardo Sáez de Villarreal

Universidad Pablo de Olavide

Physical Performance Sports Research Centre

41704 Sevilla

Spain

Email: esaesae@upo.es

ORCID: 0000-0001-9583-9287

**Table SM1.** Search strategy

| **Database** | **Full search strategy** |
| --- | --- |
| **PubMed** | ((“Cyclist*”[Title] OR “Cycling”[Title] OR “Bicycle”[Title] OR “Biking”[Title] OR “Road cycling”[Title] OR “Mountain-biking”[Title] OR “Cyclo-cross”[Title]) AND (“Resistance Training”[Mesh] OR "Plyometric Exercise"[Mesh] OR "Weight Lifting"[Mesh] OR “Strength training”[Title] OR “Weight training” [Title] OR “Resistance training”[Title] OR “Resistance exercise*”[Title] OR “Concurrent training”[Title] OR “Hypertrophy”[Title] OR “Exercise program*”[Title] OR “Weight-bearing”[Title] OR “Muscular strength”[Title] OR “Muscle strength”[Title] OR “Isoinertial”[Title] OR “Plyometric”[Title] OR “Stretch shortening exercise*”[Title] OR “Shortening contraction”[Title] OR “Reactive strength”[Title] OR “Power training”[Title] OR “Weightlifting”[Title] OR “Weight lifting”[Title] OR “Explosive strength”[Title] OR “Ballistic”[Title] OR “Local muscular”[Title] OR “TRX suspension training”[Title] OR “Suspension training”[Title] OR “Free weight training”[Title] OR “Strengthening”[Title] OR “Flywheel”[Title] OR “Eccentric training”[Title] OR “Eccentric contraction”[Title] OR “Eccentric exercis*”[Title] OR “Eccentric-weight”[Title] OR “Eccentric load*”[Title] OR “Negative muscle work”[Title])) AND (("Oxygen Consumption"[Mesh] OR "Adaptation, Physiological"[Mesh] OR “Maximal oxygen*” OR “Maximum oxygen*” OR “Peak oxygen*” OR “VO2max” OR “VO2peak” OR “Aerobic power” OR “Aerobic capacity” OR “Aerobic endurance” OR “Endurance performance” OR “Cardiovascular performance” OR “Cardiorespiratory fitness”) AND/OR (“Power at maximal oxygen*” OR “Maximal aerobic power” OR “Maximum aerobic power” OR “Maximal aerobic power” OR “Maximum aerobic power”) AND/OR (“Energy cost” OR “Energetic cost” OR “Caloric cost” OR “Metabolic cost” OR “Mechanical efficiency” OR “Energy cost of cycling” OR “Gross efficiency” OR “Cycling efficiency” OR “Gross mechanical efficiency” OR “Cycling economy” OR “Work economy” OR “Delta efficiency” OR “Net efficiency” OR “Gross muscular efficiency” OR “Muscular efficiency” OR “Work efficiency”) AND/OR ("Anaerobic Threshold"[Mesh] OR “Anaerobic threshold” OR “Ventilatory threshold” OR “Gas exchange threshold” OR “Lactate threshold” OR “Lactate” OR “Lactate turnpoint” OR “Lactate minimum power” OR “Maximal lactate steady state” OR “Onset of blood lactate accumulation” OR “Respiratory compensation point” OR “Critical power” OR “Functional threshold power” OR “metabolic threshold”) AND/OR (“Maximal anaerobic capacity” OR “Anaerobic” OR “Anaerobic power” OR “Anaerobic capacity” OR “Maximal power”) AND/OR (“Physical endurance”[Mesh] OR “Performance” OR “Time trial” OR “Time-trial” OR “race performance” OR “Distance race*” OR “Time to exhaustion” OR “Durability” OR “Stamina”)) |
| **Web of Sciences (all databases)** | ((TI=(“Cyclist*” OR “Cycling” OR “Bicycle” OR “Biking” OR “Road cycling” OR “Mountain-biking” OR “Cyclo-cross”)) AND TI=(“Strength training” OR “Weight training” OR “Resistance training” OR “Resistance exercise*” OR “Concurrent training” OR “Hypertrophy” OR “Exercise program*” OR “Weight-bearing” OR “Muscular strength” OR “Muscle strength” OR “Isoinertial” OR “Plyometric” OR “Stretch shortening exercise*” OR “Shortening contraction” OR “Reactive strength” OR “Power training” OR “Weightlifting” OR “Weight lifting” OR “Explosive strength” OR “Ballistic” OR “Local muscular” OR “TRX suspension training” OR “Suspension training” OR “Free weight training” OR “Strengthening” OR “Flywheel” OR “Eccentric training” OR “Eccentric contraction” OR “Eccentric exercis*” OR “Eccentric-weight” OR “Eccentric load*” OR “Negative muscle work”)) AND TS= (“Maximal oxygen*” OR “Maximum oxygen*” OR “Peak oxygen*” OR “VO2max” OR “VO2peak” OR “Aerobic power” OR “Aerobic capacity” OR “Aerobic endurance” OR “Endurance performance” OR “Cardiovascular performance” OR “Cardiorespiratory fitness” OR “Power at maximal oxygen*” OR “Maximal aerobic velocity” OR “Maximum aerobic power” OR “Maximal aerobic power” OR “Maximum aerobic power” OR “Energy cost” OR “Energetic cost” OR “Caloric cost” OR “Metabolic cost” OR “Mechanical efficiency” OR “Energy cost of cycling” OR “Gross efficiency” OR “Cycling efficiency” OR “Gross mechanical efficiency” OR “Cycling economy” OR “Work economy” OR “Delta efficiency” OR “Net efficiency” OR “Gross muscular efficiency” OR “Muscular efficiency” OR “Work efficiency” OR "Anaerobic Threshold" OR “Anaerobic threshold” OR “Ventilatory threshold” OR “Gas exchange threshold” OR “Lactate threshold” OR “Lactate” OR “Lactate turnpoint” OR “Lactate minimum power” OR “Maximal lactate steady state” OR “Onset of blood lactate accumulation” OR “Respiratory compensation point” OR “Critical power” OR “Functional threshold power” OR “metabolic threshold” OR “Maximal anaerobic capacity” OR “Anaerobic” OR “Anaerobic power” OR “Anaerobic capacity” OR “Maximal power” OR “Physical endurance” OR “Performance” OR “Time trial” OR “Time-trial” OR “race performance” OR “Distance race*” OR “Time to exhaustion” OR “Durability” OR “Stamina”) |
| **Scopus** | TITLE (“Cyclist*” OR “Cycling” OR “Bicycle” OR “Biking” OR “Road cycling” OR “Mountain-biking” OR “Cyclo-cross”) AND TITLE (“Strength training” OR “Weight training” OR “Resistance training” OR “Resistance exercise*” OR “Concurrent training” OR “Hypertrophy” OR “Exercise program*” OR “Weight-bearing” OR “Muscular strength” OR “Muscle strength” OR “Isoinertial” OR “Plyometric” OR “Stretch shortening exercise*” OR “Shortening contraction” OR “Reactive strength” OR “Power training” OR “Weightlifting” OR “Weight lifting” OR “Explosive strength” OR “Ballistic” OR “Local muscular” OR “TRX suspension training” OR “Suspension training” OR “Free weight training” OR “Strengthening” OR “Flywheel” OR “Eccentric training” OR “Eccentric contraction” OR “Eccentric exercis*” OR “Eccentric-weight” OR “Eccentric load*” OR “Negative muscle work”) AND ALL (“Maximal oxygen*” OR “Maximum oxygen*” OR “Peak oxygen*” OR “VO2max” OR “VO2peak” OR “Aerobic power” OR “Aerobic capacity” OR “Aerobic endurance” OR “Endurance performance” OR “Cardiovascular performance” OR “Cardiorespiratory fitness” OR “Power at maximal oxygen*” OR “Maximal aerobic velocity” OR “Maximum aerobic power” OR “Maximal aerobic power” OR “Maximum aerobic power” OR “Energy cost” OR “Energetic cost” OR “Caloric cost” OR “Metabolic cost” OR “Mechanical efficiency” OR “Energy cost of cycling” OR “Gross efficiency” OR “Cycling efficiency” OR “Gross mechanical efficiency” OR “Cycling economy” OR “Work economy” OR “Delta efficiency” OR “Net efficiency” OR “Gross muscular efficiency” OR “Muscular efficiency” OR “Work efficiency” OR "Anaerobic Threshold" OR “Anaerobic threshold” OR “Ventilatory threshold” OR “Gas exchange threshold” OR “Lactate threshold” OR “Lactate” OR “Lactate turnpoint” OR “Lactate minimum power” OR “Maximal lactate steady state” OR “Onset of blood lactate accumulation” OR “Respiratory compensation point” OR “Critical power” OR “Functional threshold power” OR “metabolic threshold” OR “Maximal anaerobic capacity” OR “Anaerobic” OR “Anaerobic power” OR “Anaerobic capacity” OR “Maximal power” OR “Physical endurance” OR “Performance” OR “Time trial” OR “Time-trial” OR “race performance” OR “Distance race*” OR “Time to exhaustion” OR “Durability” OR “Stamina”) |

Mesh: Medical Subject Headings; TI: title; TS: topic.

**Table SM2.** Reasons for exclusion of the reports in the review

| **Reason for exclusion** | **Studies** |
| --- | --- |
| Absence of heavy strength training | Bastiaans et al. 2001  Psilander et al. 2015 |
| Non-participation of cyclists | Moysi et al. 1998  Hansen et al. 2007  Minahan and Wood 2008  Zoladz et al. 2012  McNamara and Stearne 2013  Silva et al. 2022 |
| Lack of a comparison group | Paton and Hopkins 2005  Koninckx et al. 2010  Louis et al. 2012  Kristoffersen et al. 2019  Gil-Cabrera et al. 2021  Montalvo-Pérez et al. 2021  Valenzuela et al. 2021  Bláfoss et al. 2022  Jones et al. 2022 |
| Repeated results | Hansen et al. 2012  Rønnestad et al. 2012  Rønnestad et al. 2017  Vikmoen et al. 2017 |

**Table SM3.** Physiotherapy Evidence Database (PEDro) scale.

| **Author** | **N1** | **N2** | **N3** | **N4** | **N5** | **N6** | **N7** | **N8** | **Total** | **Risk of bias** |
| --- | --- | --- | --- | --- | --- | --- | --- | --- | --- | --- |
| Bishop et al. 1999 | Yes | 1 | 0 | 1 | 1 | 1 | 1 | 1 | 6 | Low risk |
| Jackson et al. 2007 | Yes | 1 | 0 | 1 | 1 | 1 | 1 | 1 | 6 | Low risk |
| Levin et al. 2009 | Yes | 1 | 0 | 1 | 0 | 1 | 1 | 1 | 5 | Moderate risk |
| Hausswirth et al. 2010 | Yes | 1 | 0 | 1 | 1 | 1 | 1 | 1 | 6 | Low risk |
| Rønnestad et al. 2010a | Yes | 0 | 0 | 1 | 1 | 1 | 1 | 1 | 5 | Moderate risk |
| Rønnestad et al. 2010b | Yes | 1 | 0 | 0 | 1 | 1 | 1 | 1 | 5 | Moderate risk |
| Sunde et al. 2010 | Yes | 1 | 0 | 1 | 1 | 1 | 1 | 1 | 6 | Low risk |
| Aagaard et al. 2011 | Yes | 1 | 0 | 1 | 1 | 1 | 1 | 1 | 6 | Low risk |
| Rønnestad et al. 2011 | Yes | 0 | 0 | 1 | 1 | 1 | 1 | 1 | 5 | Moderate risk |
| Rønnestad et al. 2015 | Yes | 1 | 0 | 1 | 1 | 1 | 1 | 1 | 6 | Low risk |
| Rønnestad et al. 2016 | Yes | 1 | 0 | 1 | 1 | 1 | 1 | 1 | 6 | Low risk |
| Vikmoen et al. 2016 | Yes | 1 | 0 | 1 | 0 | 1 | 1 | 1 | 5 | Moderate risk |
| Beattie et al. 2017 | Yes | 0 | 0 | 1 | 1 | 1 | 1 | 1 | 5 | Moderate risk |
| Del Vecchio et al. 2019 | Yes | 0 | 0 | 1 | 1 | 1 | 1 | 1 | 5 | Moderate risk |
| Luckin-Baldwin et al. 2021 | Yes | 1 | 1 | 0 | 1 | 1 | 1 | 1 | 6 | Low risk |
| Ji et al. 2022 | Yes | 0 | 0 | 1 | 1 | 1 | 1 | 1 | 5 | Moderate risk |
| Sitko et al. 2024 | Yes | 1 | 1 | 0 | 1 | 1 | 1 | 1 | 6 | Low risk |

N1: eligibility criteria were specified; N2: subjects were randomly allocated to groups; N3: allocation was concealed; N4: the groups were similar at baseline regarding the most important prognostic indicators; N5: measures of at least one key outcome were obtained from more than 85% of the subjects initially allocated to groups; N6: all subjects for whom outcome measures were available received the treatment or control condition as allocated or, where this was not the case, data for at least one key outcome was analysed by “intention to treat”; N7: the results of between-group statistical comparisons are reported for at least one key outcome; N8: the study provides both point measures and measures of variability for at least one key outcome; Risk of bias: ≥ 6 points = “low risk”, 4 to 5 points = “moderate risk”, and ≤ 3 points = “high risk”. Items 5 to 7 of the original scale were removed due to the infrequency of blinding of subjects, evaluators, and researchers in supervised exercise interventions.

**Table SM4.** GRADE assessment for the certainty of evidence.

| **Certainty assessment** | | | | | | **Nº of participants** | | **Certainty** |
| --- | --- | --- | --- | --- | --- | --- | --- | --- |
| **Nº of studies** | **Risk of bias** | **Inconsistency** | **Indirectness** | **Imprecision** | **Risk of publication bias** | **Experimental group** | **Control group** |  |
| **VO_2_max (follow-up: mean 13.07 weeks)** | | | | | | | | |
| 12 | serious^a^ | not serious | not serious | serious^b^ | not serious | 109 | 83 | Low |
| **pVO_2_max (follow-up: mean 13.27 weeks)** | | | | | | | | |
| 10 | serious^a^ | not serious | not serious | serious^b^ | not serious | 89 | 71 | Low |
| **Maximum metabolic steady state (follow-up: mean 13.00 weeks)** | | | | | | | | |
| 8 | serious^a^ | not serious | not serious | serious^b^ | not serious | 76 | 55 | Low |
| **Cycling efficiency (follow-up: mean 14.91 weeks)** | | | | | | | | |
| 10 | serious^a^ | not serious | not serious | serious^b^ | not serious | 92 | 75 | Low |
| **Anaerobic capacity (follow-up: mean 15.00 weeks)** | | | | | | | | |
| 6 | serious^a^ | not serious | not serious | serious^b^ | not serious | 60 | 43 | Low |
| **Anaerobic power (follow-up: mean 15.22 weeks)** | | | | | | | | |
| 8 | serious^a^ | not serious | not serious | serious^b^ | not serious | 78 | 64 | Low |
| **Cycling performance (follow-up: mean 14.10 weeks)** | | | | | | | | |
| 9 | serious^a^ | not serious | not serious | serious^b^ | not serious | 92 | 67 | Low |

a. Downgraded by one level because the median PEDro scale is < 6.

b. Downgraded by one level because n < 800 and/or the 95% confidence interval crossed the small effect size.

**Table SM5.** Results of meta-regression and subgroup analyses in search of possible moderators of heavy strength training on VO_2_max.

| **VO_2_max** | *n* groups | *β_0_* Hedges' *g* (SE) | *β_0_* CI 95% | *t_0_*(*df*)*, p* value | *β_1_* Hedges' *g* (SE) | *β_1_* CI 95% | *t_1_*(*df*)*, p* value | *F*(*df_1_, df_2_*)*, p* value |
| --- | --- | --- | --- | --- | --- | --- | --- | --- |
| Subject characteristics |  |  |  |  |  |  |  |  |
| Sex | 13 |  |  |  |  |  |  | *F*(2,10) = 0.079, *p* = 0.924 |
| Male | 6 | -0.012 (0.205) | -0.469 to 0.446 | *t*(10) = -0.057, *p* = 0.956 |  |  |  |  |
| Female | 2 | 0.021 (0.314) | -0.679 to 0.720 | *t*(10) = 0.066, *p* = 0.949 | 0.032 (0.375) | -0.804 to 0.869 | *t*(10) = 0.086, *p* = 0.933 |  |
| Male-Female | 5 | -0.116 (0.238) | -0.647 to 0.414 | *t*(10) = -0.488, *p* = 0.636 | -0.104 (0.314) | -0.805 to 0.596 | *t*(10) = -0.332, *p* = 0.747 |  |
| Age | 13 | 0.204 (0.868) | -1.706 to 2.115 | *t*(11) = 0.236, *p* = 0.818 | -0.008 (0.029) | -0.073 to 0.056 | *t*(11) = -0.287, *p* = 0.780 | *F*(1,11) = 0.082, *p* = 0.780 |
| Body mass | 11 | 0.088 (1.983) | -4.397 to 4.574 | *t*(9) = 0.045, *p* = 0.965 | -0.002 (0.028) | -0.065 to 0.062 | *t*(9) = -0.066, *p* = 0.949 | *F*(1,9) = 0.004, *p* = 0.949 |
| Height | 10 | -1.796 (7.286) | -18.597 to 15.005 | *t*(8) = -0.247, *p* = 0.811 | 0.010 (0.041) | -0.084 to 0.105 | *t*(8) = 0.248, *p* = 0.810 | *F*(1,8) = 0.062, *p* = 0.810 |
| Initial VO_2_max | 13 | -0.265 (1.078) | -2.638 to 2.109 | *t*(11) = -0.245, *p* = 0.811 | 0.004 (0.017) | -0.034 to 0.042 | *t*(11) = 0.209, *p* = 0.838 | *F*(1,11) = 0.044, *p* = 0.838 |
| Strength training intervention |  |  |  |  |  |  |  |  |
| Weeks | 13 | 0.023 (0.326) | -0.693 to 0.740 | *t*(11) = 0.071, *p* = 0.944 | -0.005 (0.022) | -0.054 to 0.044 | *t*(11) = -0.219, *p* = 0.831 | *F*(1,11) = 0.048, *p* = 0.831 |
| Sessions per week | 13 | -0.072 (0.573) | -1.334 to 1.190 | *t*(11) = -0.125, *p* = 0.903 | 0.014 (0.248) | -0.533 to 0.560 | *t*(11) = 0.055, *p* = 0.957 | *F*(1,11) = 0.003, *p* = 0.957 |
| Number of total sessions | 13 | 0.285 (0.490) | -0.821 to 1.336 | *t*(11) = 0.526, *p* = 0.609 | -0.011 (0.018) | -0.050 to 0.028 | *t*(11) = -0.636, *p* = 0.538 | *F*(1,11) = 0.405, *p* = 0.538 |

In the subgroup analysis (categorical variables), the first variable of the category was considered as the reference. *β_0_*, intercept; *β_1_*, regression coefficient; n groups, number of experimental groups; CI, confidence Interval; df, degree freedom; SE, standard error; *t_0,_* t-value associated with the intercept, *t_0,_* t-value associated with the regression coefficient.

**Table SM6.** Results of meta-regression and subgroup analyses in search of possible moderators of heavy strength training on power related to VO_2_max.

| **pVO_2_max** | *n* groups | *β_0_* Hedges' *g* (SE) | *β_0_* CI 95% | *t_0_*(*df*)*, p* value | *β_1_* Hedges' *g* (SE) | *β_1_* CI 95% | *t_1_*(*df*)*, p* value | *F*(*df_1_, df_2_*)*, p* value |
| --- | --- | --- | --- | --- | --- | --- | --- | --- |
| Subject characteristics |  |  |  |  |  |  |  |  |
| Sex | 11 |  |  |  |  |  |  | *F*(2,8) = 0.192, *p* = 0.829 |
| Male | 5 | 0.259 (0.222) | -0.254 to 0.772 | *t*(8) = 1.163, *p* = 0.278 |  |  |  |  |
| Female | 1 | 0.001 (0.444) | -1.024 to 1.024 | *t*(8) = 0.001, *p* = 0.999 | -0.259 (0.469) | -1.404 to 0.886 | *t*(8) = 0.521, *p* = 0.616 |  |
| Male-Female | 5 | 0.102 (0.239) | -0.449 to 0.654 | *t*(8) = 0.429, *p* = 0.679 | -0.156 (0.326) | -0.909 to 0.597 | *t*(8) = -0.479, *p* = 0.645 |  |
| Age | 11 | 0.790 (0.663) | -0.709 to 2.289 | *t*(9) = 1.192, *p* = 0.264 | -0.019 (0.020) | -0.065 to 0.026 | *t*(9) = -0.971, *p* = 0.357 | *F*(1,9) = 0.943, *p* = 0.357 |
| Body mass | 9 | 2.231 (2.488) | -3.652 to 8.113 | *t*(7) = 0.897, *p* = 0.400 | -0.028 (0.034) | -0.109 to 0.052 | *t*(7) = -0.824, *p* = 0.437 | *F*(1,7) = 0.679, *p* = 0.437 |
| Height | 9 | -1.831 (7.237) | -18.944 to 15.282 | *t*(7) = -0.253, *p* = 0.808 | 0.012 (0.041) | -0.084 to 0.107 | *t*(7) = 0.283, *p* = 0.785 | *F*(1,7) = 0.080, *p* = 0.785 |
| Initial VO_2_max | 10 | -1.785 (1.448) | -5.123 to 1.554 | *t*(8) = -1.233, *p* = 0.253 | 0.032 (0.023) | -0.021 to 0.085 | *t*(8) = 1.391, *p* = 0.202 | *F*(1,8) = 1.935, *p* = 0.202 |
| Strength training intervention |  |  |  |  |  |  |  |  |
| Weeks | 11 | -0.298 (0.346) | -1.081 to 0.484 | *t*(9) = -0.863, *p* = 0.411 | 0.035 (0.023) | -0.018 to 0.088 | *t*(9) = 1.490, *p* = 0.170 | *F*(1,9) = 2.221, *p* = 0.170 |
| Sessions per week | 11 | 0.811 (0.611) | -0.572 to 2.194 | *t*(9) = 1.326, *p* = 0.217 | -0.299 (0.274) | -0.919 to 0.320 | *t*(9) = -1.093, *p* = 0.303 | *F*(1,9) = 1.194, *p* = 0.303 |
| Number of total sessions | 11 | -0.474 (0.545) | -1.707 to 0.759 | *t*(9) = -0.870, *p* = 0.407 | 0.025 (0.020) | -0.021 to 0.071 | *t*(9) = 1.220, *p* = 0.254 | *F*(1,9) = 1.488, *p* = 0.254 |

In the subgroup analysis (categorical variables), the first variable of the category was considered as the reference. *β_0_*, intercept; *β_1_*, regression coefficient; n groups, number of experimental groups; CI, confidence Interval; df, degree freedom; pVO_2_max, power related to VO_2_max; SE, standard error; *t_0,_* t-value associated with the intercept, *t_0,_* t-value associated with the regression coefficient.

**Table SM7.** Results of meta-regression and subgroup analyses in search of possible moderators of heavy strength training on maximum metabolic steady state.

| **MMSS** | *n* groups | *β_0_* Hedges' *g* (SE) | *β_0_* CI 95% | *t_0_*(*df*)*, p* value | *β_1_* Hedges' *g* (SE) | *β_1_* CI 95% | *t_1_*(*df*)*, p* value | *F*(*df_1_, df_2_*)*, p* value |
| --- | --- | --- | --- | --- | --- | --- | --- | --- |
| Subject characteristics |  |  |  |  |  |  |  |  |
| Sex | 9 |  |  |  |  |  |  | *F*(2,6) = 0.148, *p* = 0.865 |
| Male | 4 | 0.032 (0.251) | -0.582 to 0.646 | *t*(6) = 0.127, *p* = 0.903 |  |  |  |  |
| Female | 2 | 0.208 (0.315) | -0.562 to 0.979 | *t*(6) = 0.661, *p* = 0.533 | 0.176 (0.403) | -0.809 to 1.162 | *t*(6) = 0.438, *p* = 0.677 |  |
| Male-Female | 3 | -0.026 (0.339) | -0.856 to 0.803 | *t*(6) = -0.077, *p* = 0.941 | -0.058 (0.422) | -1.090 to 0.974 | *t*(6) = -0.138, *p* = 0.895 |  |
| Age | 9 | -0.055 (0.898) | -2.180 to 2.069 | *t*(7) = -0.062, *p* = 0.953 | 0.004 (0.030) | -0.067 to 0.075 | *t*(7) = -0.062, *p* = 0.953 | *F*(1,7) = 0.020, *p* = 0.892 |
| Body mass | 9 | 1.449 (2.351) | -4.110 to 7.008 | *t*(7) = 0.616, *p* = 0.557 | -0.020 (0.034) | -0.100 to 0.060 | *t*(7) = -0.589, *p* = 0.575 | *F*(1,7) = 0.346, *p* = 0.575 |
| Height | 8 | 4.397 (7.844) | -14.795 to 23.590 | *t*(6) = 0.561, *p* = 0.595 | -0.025 (0.044) | -0.133 to 0.084 | *t*(6) = -0.555, *p* = 0.599 | *F*(1,6) = 0.308, *p* = 0.599 |
| Initial VO_2_max | 9 | 0.305 (1.200) | -2.533 to 3.143 | *t*(7) = 0.254, *p* = 0.807 | -0.004 (0.019) | -0.049 to 0.042 | *t*(7) = -0.199, *p* = 0.848 | *F*(1,7) = 0.040, *p* = 0.848 |
| Strength training intervention |  |  |  |  |  |  |  |  |
| Weeks | 9 | 0.097 (0.415) | -0.885 to 1.079 | *t*(7) = 0.234, *p* = 0.822 | -0.002 (0.029) | -0.070 to 0.066 | *t*(7) = -0.075, *p* = 0.943 | *F*(1,7) = 0.006, *p* = 0.943 |
| Sessions per week | 9 | 0.349 (0.745) | -1.412 to 2.109 | *t*(7) = 0.468, *p* = 0.654 | -0.128 (0.331) | -0.910 to 0.654 | *t*(7) = -0.386, *p* = 0.711 | *F*(1,7) = 0.149, *p* = 0.711 |
| Number of total sessions | 9 | 0.506 (0.563) | -0.825 to 1.838 | *t*(7) = 0.900, *p* = 0.398 | -0.016 (0.020) | -0.064 to 0.031 | *t*(7) = -0.816, *p* = 0.442 | *F*(1,7) = 0.665, *p* = 0.442 |

In the subgroup analysis (categorical variables), the first variable of the category was considered as the reference. *β_0_*, intercept; *β_1_*, regression coefficient; n groups, number of experimental groups; CI, confidence Interval; df, degree freedom; MMSS, maximum metabolic steady state; SE, standard error; *t_0,_* t-value associated with the intercept, *t_0,_* t-value associated with the regression coefficient.

**Table SM8.** Results of meta-regression and subgroup analyses in search of possible moderators of heavy strength training on anaerobic capacity.

| **Anaerobic capacity** | *n* groups | *β_0_* Hedges' *g* (SE) | *β_0_* CI 95% | *t_0_*(*df*)*, p* value | *β_1_* Hedges' *g* (SE) | *β_1_* CI 95% | *t_1_*(*df*)*, p* value | *F*(*df_1_, df_2_*)*, p* value |
| --- | --- | --- | --- | --- | --- | --- | --- | --- |
| Subject characteristics |  |  |  |  |  |  |  |  |
| Sex | 7 |  |  |  |  |  |  | *F*(2,4) = 0.210, *p* = 0.819 |
| Male | 4 | 0.114 (0.337) | -0.823 to 1.050 | *t*(4) = 0.337, *p* = 0.753 |  |  |  |  |
| Female | 1 | 0.478 (0.451) | -0.773 to 1.729 | *t*(4) = 1.060, *p* = 0.349 | 0.364 (0.563) | -1.199 to 1.927 | *t*(4) = 0.647, *p* = 0.553 |  |
| Male-Female | 2 | 0.226 (0.268) | -0.518 to 0.970 | *t*(4) = 0.843, *p* = 0.447 | 0.112 (0.431) | -1.084 to 1.308 | *t*(4) = 0.261, *p* = 0.807 |  |
| Age | 7 | 0.376 (0.682) | -1.377 to 2.130 | *t*(5) = 0.551, *p* = 0.605 | -0.004 (0.020) | -0.057 to 0.048 | *t*(5) = -0.215, *p* = 0.838 | *F*(1,5) = 0.046, *p* = 0.838 |
| Body mass | 6 | 1.763 (2.725) | -5.803 to 9.328 | *t*(4) = 0.647, *p* = 0.553 | -0.020 (0.037) | -0.124 to 0.083 | *t*(4) = -0.550, *p* = 0.612 | *F*(1,4) = 0.302, *p* = 0.612 |
| Height | 6 | 4.511 (7.586) | -16.550 to 25.572 | *t*(4) = 0.595, *p* = 0.584 | -0.024 (0.042) | -0.142 to 0.094 | *t*(4) = -0.563, *p* = 0.604 | *F*(1,4) = 0.317, *p* = 0.604 |
| Initial VO_2_max | 6 | 1.338 (1.792) | -3.636 to 6.313 | *t*(4) = 0.747, *p* = 0.497 | -0.017 (0.028) | -0.094 to 0.061 | *t*(4) = -0.599, *p* = 0.581 | *F*(1,4) = 0.359, *p* = 0.581 |
| Strength training intervention |  |  |  |  |  |  |  |  |
| Weeks | 7 | 0.467 (0.499) | -0.816 to 1.749 | *t*(5) = 0.935, *p* = 0.393 | -0.015 (0.030) | -0.093 to 0.063 | *t*(5) = -0.502, *p* = 0.637 | *F*(1,5) = 0.252, *p* = 0.637 |
| Sessions per week | 7 | -0.238 (1.249) | -3.449 to 2.974 | *t*(5) = -0.190, *p* = 0.857 | 0.259 (0.677) | -1.480 to 1.998 | *t*(5) = 0.383, *p* = 0.718 | *F*(1,5) = 0.146, *p* = 0.718 |
| Number of total sessions | 7 | 0.763 (0.882) | -1.504 to 3.030 | *t*(5) = 0.865, *p* = 0.426 | -0.020 (0.033) | -0.106 to 0.065 | *t*(5) = -0.613, *p* = 0.567 | *F*(1,5) = 0.376, *p* = 0.567 |

In the subgroup analysis (categorical variables), the first variable of the category was considered as the reference. *β_0_*, intercept; *β_1_*, regression coefficient; n groups, number of experimental groups; CI, confidence Interval; df, degree freedom; SE, standard error; *t_0,_* t-value associated with the intercept, *t_0,_* t-value associated with the regression coefficient.

**Table SM9.** Results of meta-regression and subgroup analyses in search of possible moderators of heavy strength training on anaerobic power.

| **Anaerobic power** | *n* groups | *β_0_* Hedges' *g* (SE) | *β_0_* CI 95% | *t_0_*(*df*)*, p* value | *β_1_* Hedges' *g* (SE) | *β_1_* CI 95% | *t_1_*(*df*)*, p* value | *F*(*df_1_, df_2_*)*, p* value |
| --- | --- | --- | --- | --- | --- | --- | --- | --- |
| Subject characteristics |  |  |  |  |  |  |  |  |
| Sex | 9 |  |  |  |  |  |  | F(2,6) = 0.220, p = 0.808 |
| Male | 4 | 0.714 (0.329) | -0.090 to 1.519 | *t*(6) = 2.173, *p* = 0.073 |  |  |  |  |
| Female | 1 | 0.476 (0.630) | -1.066 to 2.019 | t(6) = 0.756, p = 0.478 | -0.238 (0.711) | -1.978 to 1.501 | t(6) = -0.335, p = 0.749 |  |
| Male-Female | 4 | 0.401 (0.352) | -0.461 to 1.264 | t(6) = 1.138, p = 0.298 | -0.313 (0.482) | -1.493 to 0.866 | t(6) = -0.650, p = 0.540 |  |
| Age | 9 | 1.267 (0.810) | -0.648 to 3.183 | t(7) =1.565, p = 0.162 | -0.022 (0.024) | -0.079 to 0.035 | t(7) = -0.902, p = 0.397 | F(1,7) = 0.814, p = 0.397 |
| Body mass | 8 | 4.300 (3.314) | -3.810 to 12.411 | t(6) = 1.297, p = 0.242 | -0.051 (0.046) | -0.163 to 0.061 | t(6) = -1.123, p = 0.304 | F(1,6) = 1.262, p = 0.304 |
| Height | 8 | 2.998 (10.683) | -23.143 to 29.140 | t(6) = 0.281, p = 0.788 | -0.014 (0.060) | -0.160 to 0.132 | t(6) = -0.229, p = 0.827 | F(1,6) = 0.052, p = 0.827 |
| Initial VO_2_max | 7 | 0.770 (1.770) | -3.780 to 5.320 | t(5) = 0.435, p = 0.682 | -0.005 (0.028) | -0.076 to 0.066 | t(5) = -0.176, p = 0.867 | F(1,5) = 0.031, p = 0.867 |
| Strength training intervention |  |  |  |  |  |  |  |  |
| Weeks | 9 | 0.743 (0.590) | -0.651 to 2.137 | t(7) = 1.260, p = 0.248 | -0.012 (0.036) | -0.097 to 0.073 | t(7) = -0.335, p = 0.747 | F(1,7) = 0.112, p = 0.747 |
| Sessions per week | 9 | -0.380 (1.522) | -3.980 to 3.220 | t(7) = -0.250, p = 0.810 | 0.504 (0.809) | -1.409 to 2.416 | t(7) = 0.622, p = 0.553 | F(1,7) = 0.387, p = 0.553 |
| Number of total sessions | 9 | 0.549 (0.870) | -1.509 to 2.607 | t(7) = 0.631, p = 0.548 | 0.001 (0.031) | -0.073 to 0.074 | t(7) = 0.011, p = 0.992 | F(1,7) = 0.001, p = 0.992 |

In the subgroup analysis (categorical variables), the first variable of the category was considered as the reference. *β_0_*, intercept; *β_1_*, regression coefficient; n groups, number of experimental groups; CI, confidence Interval; df, degree freedom; SE, standard error; *t_0,_* t-value associated with the intercept, *t_0,_* t-value associated with the regression coefficient.

**Table SM10.** Results of meta-regression and subgroup analyses in search of possible moderators of heavy strength training on cycling efficiency.

| **Cycling efficiency** | *n* groups | *n* ES | *β_0_* Hedges' *g* (SE) | *β_0_* CI 95% | *t_0_*(*df*)*, p* value | *β_1_* Hedges' *g* (SE) | *β_1_* CI 95% | *t_1_*(*df*)*, p* value | *F*(*df_1_, df_2_*)*, p* value |
| --- | --- | --- | --- | --- | --- | --- | --- | --- | --- |
| Subject characteristics |  |  |  |  |  |  |  |  |  |
| Sex | 10 | 17 |  |  |  |  |  |  | *F*(2,14) = 0.938, *p* = 0.414 |
| Male | 4 | 4 | -0.047 (0.256) | -0.597 to 0.503 | *t*(14) = -0.182, *p* = 0.858 |  |  |  |  |
| Female | 1 | 1 | -0.436 (0.449) | -1.400 to 0.528 | *t*(14) = -0.970, *p* = 0.349 | -0.389 (0.517) | -1.499 to 0.721 | *t*(14) = -0.752, *p* = 0.465 |  |
| Male-Female | 5 | 12 | -0.451 (0.151) | -0.775 to -0.126 | ***t*(14) = -2.980, *p* = 0.010** | -0.404 (0.298) | -1.042 to 0.235 | *t*(14) = -1.357, *p* = 0.196 |  |
| Age | 10 | 17 | 0.705 (0.806) | -1.012 to 2.422 | *t*(15) = 0.875, *p* = 0.395 | -0.034 (0.026) | -0.089 to 0.021 | *t*(15) = -1.330, *p* = 0.204 | *F*(1,15) = 1.768, *p* = 0.204 |
| Body mass | 8 | 11 | 0.484 (2.820) | -5.895 to 6.863 | *t*(9) = 0.172, *p* = 0.868 | -0.011 (0.039) | -0.099 to 0.077 | *t*(9) = -0.279, *p* = 0.786 | *F*(1,9) = 0.078, *p* = 0.786 |
| Height | 9 | 15 | 1.039 (5.970) | -11.859 to 13.937 | *t*(13) = 0.174, *p* = 0.865 | -0.008 (0.033) | -0.080 to 0.064 | *t*(13) = -0.235, *p* = 0.818 | *F*(1,13) = 0.055, *p* = 0.818 |
| Initial VO_2_max | 10 | 17 | -1.145 (1.067) | -3.420 to 1.130 | *t*(15) = -1.073, *p* = 0.300 | 0.013 (0.017) | -0.024 to 0.050 | *t*(15) = 0.747, *p* = 0.467 | *F*(1,15) = 0.558, *p* = 0.467 |
| Intensity (power) | 7 | 14 | -0.935 (0.696) | -2.452 to 0.582 | *t*(12) = -1.343, *p* = 0.204 | 0.003 (0.004) | -0.005 to 0.011 | *t*(12) = 0.763, *p* = 0.460 | *F*(1,12) = 0.582, *p* = 0.460 |
| Strength training intervention |  |  |  |  |  |  |  |  |  |
| Weeks | 10 | 17 | -0.140 (0.305) | -0.789 to 0.510 | *t*(15) = -0.458, *p* = 0.653 | -0.013 (0.017) | -0.049 to 0.023 | *t*(15) = -0.770, *p* = 0.453 | *F*(1,15) = 0.593, *p* = 0.453 |
| Sessions per week | 10 | 17 | -0.999 (0.504) | -2.073 to 0.075 | *t*(15) = -1.982, *p* = 0.066 | 0.311 (0.235) | -0.190 to 0.813 | *t*(15) = 1.323, *p* = 0.206 | *F*(1,15) = 1.749, *p* = 0.206 |
| Number of total sessions | 10 | 17 | -0.342 (0.398) | -1.190 to 0.507 | *t*(15) = -0.859, *p* = 0.404 | -0.001 (0.012) | -0.026 to 0.026 | *t*(15) = -0.031, *p* = 0.976 | *F*(1,15) = 0.001, *p* = 0.976 |

In the subgroup analysis (categorical variables), the first variable of the category was considered as the reference. *β_0_*, intercept; *β_1_*, regression coefficient; n groups, number of experimental groups; CI, confidence Interval; df, degree freedom; SE, standard error; *t_0,_* t-value associated with the intercept, *t_0,_* t-value associated with the regression coefficient. Results in bold represent a significant effect (*p* ≤ 0.05).

**Table SM11.** Results of meta-regression and subgroup analyses in search of possible moderators of heavy strength training on cycling performance.

| **Cycling performance** | *n* groups | *β_0_* Hedges' *g* (SE) | *β_0_* CI 95% | *t_0_*(*df*)*, p* value | *β_1_* Hedges' *g* (SE) | *β_1_* CI 95% | *t_1_*(*df*)*, p* value | *F*(*df_1_, df_2_*)*, p* value |
| --- | --- | --- | --- | --- | --- | --- | --- | --- |
| Subject characteristics |  |  |  |  |  |  |  |  |
| Sex | 10 |  |  |  |  |  |  | *F*(2,7) = 0.836, *p* = 0.473 |
| Male | 3 | 0.697 (0.269) | 0.061 to 1.333 | ***t*(7) = 2.593, *p* = 0.036** |  |  |  |  |
| Female | 2 | 0.161 (0.317) | -0.589 to 0.910 | *t*(7) = 0.507, *p* = 0.628 | -0.536 (0.416) | -1.520 to 0.447 | *t*(7) = -1.290, *p* = 0.238 |  |
| Male-Female | 5 | 0.449 (0.242) | -0.123 to 1.021 | *t*(7) = 1.856, *p* = 0.106 | -0.248 (0.362) | -1.104 to 0.607 | *t*(7) = -0.687, *p* = 0.514 |  |
| Age | 10 | 0.736 (0.965) | -1.490 to 2.961 | *t*(8) = 0.762, *p* = 0.468 | -0.010 (0.034) | -0.087 to 0.068 | *t*(8) = -0.286, *p* = 0.782 | *F*(1,8) = 0.082, *p* = 0.782 |
| Body mass | 9 | -1.582 (2.163) | -6.697 to 3.534 | *t*(7) = -0.731, *p* = 0.489 | 0.029 (0.031) | -0.044 to 0.103 | *t*(7) = 0.947, *p* = 0.375 | *F*(1,7) = 0.896, *p* = 0.375 |
| Height | 8 | -0.251 (7.536) | -18.689 to 18.188 | *t*(6) = -0.033, *p* = 0.975 | 0.004 (0.042) | -0.099 to 0.108 | *t*(6) = 0.107, *p* = 0.918 | *F*(1,6) = 0.011, *p* = 0.918 |
| Initial VO_2_max | 9 | -0.750 (1.208) | -3.607 to 2.107 | *t*(7) = -0.621, *p* = 0.555 | 0.018 (0.019) | -0.027 to 0.064 | *t*(7) = 0.945, *p* = 0.376 | *F*(1,7) = 0.893, *p* = 0.376 |
| Strength training intervention |  |  |  |  |  |  |  |  |
| Weeks | 10 | 0.379 (0.427) | -0.606 to 1.365 | *t*(8) = 0.887, *p* = 0.401 | 0.006 (0.028) | -0.059 to 0.071 | *t*(8) = 0.210, *p* = 0.839 | *F*(1,8) = 0.044, *p* = 0.839 |
| Sessions per week | 10 | 0.760 (0.757) | -0.986 to 2.507 | *t*(8) = 1.004, *p* = 0.345 | -0.147 (0.367) | -0.994 to 0.699 | *t*(8) = -0.401, *p* = 0.699 | *F*(1,8) = 0.161, *p* = 0.699 |
| Number of total sessions | 10 | 0.396 (0.676) | -1.162 to 1.955 | *t*(8) = 0.586, *p* = 0.574 | 0.002 (0.025) | -0.054 to 0.059 | *t*(8) = 0.101, *p* = 0.922 | *F*(1,8) = 0.010, *p* = 0.922 |

In the subgroup analysis (categorical variables), the first variable of the category was considered as the reference. *β_0_*, intercept; *β_1_*, regression coefficient; n groups, number of experimental groups; CI, confidence Interval; df, degree freedom; SE, standard error; *t_0,_* t-value associated with the intercept, *t_0,_* t-value associated with the regression coefficient. Results in bold represent a significant effect (*p* ≤ 0.05).

**Fig. SM1** Funnel plot of studies that implemented heavy strength training and analysed related outcomes.


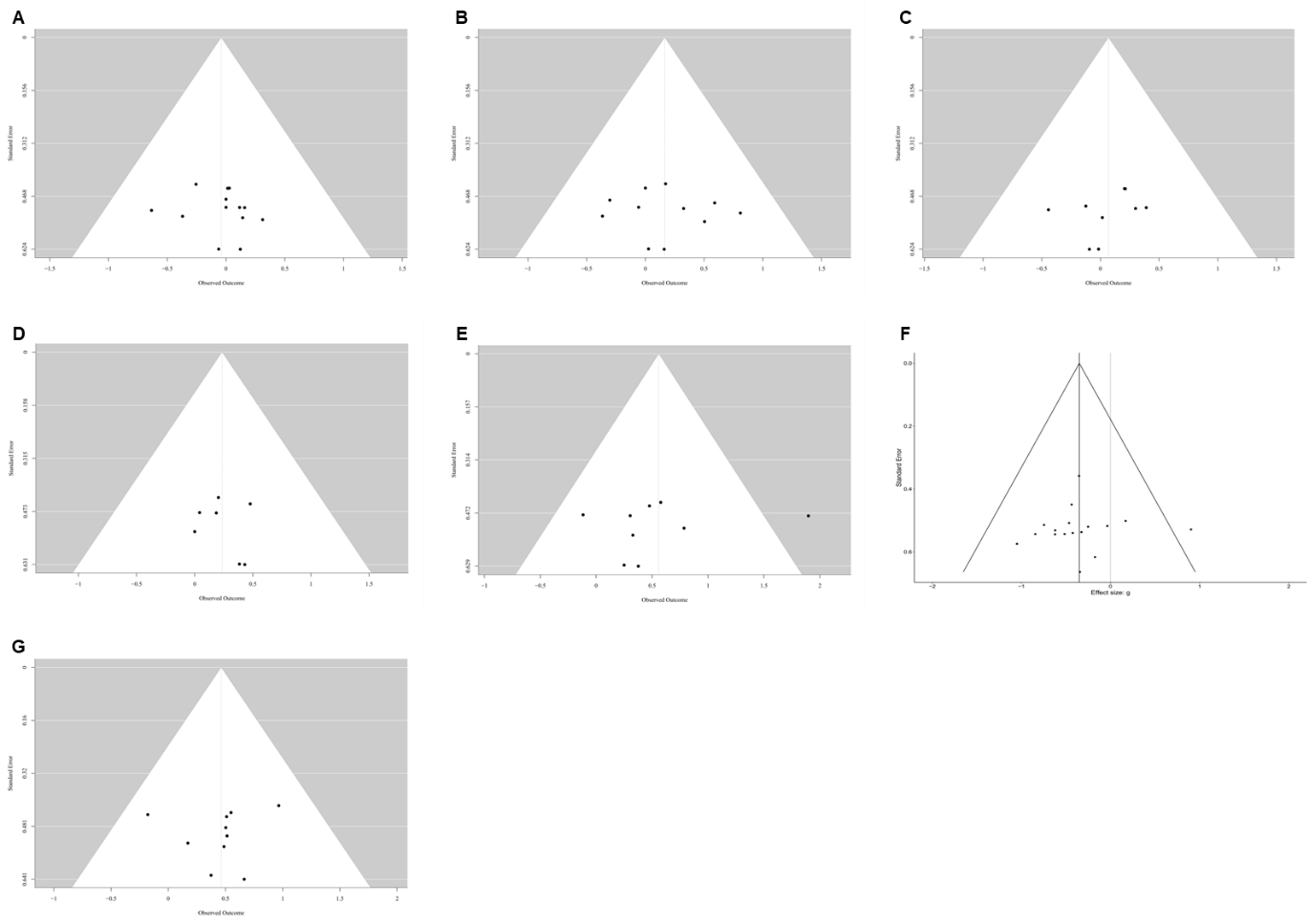


A, maximal oxygen uptake (VO_2_max); B, power related to VO_2_max (pVO_2_max); C, maximal metabolic steady state (MMSS); D, anaerobic capacity; E, anaerobic power; F, cycling efficiency; G, cycling performance.

**Fig. SM2** Forest plot of the effect of heavy strength training on VO_2_max.


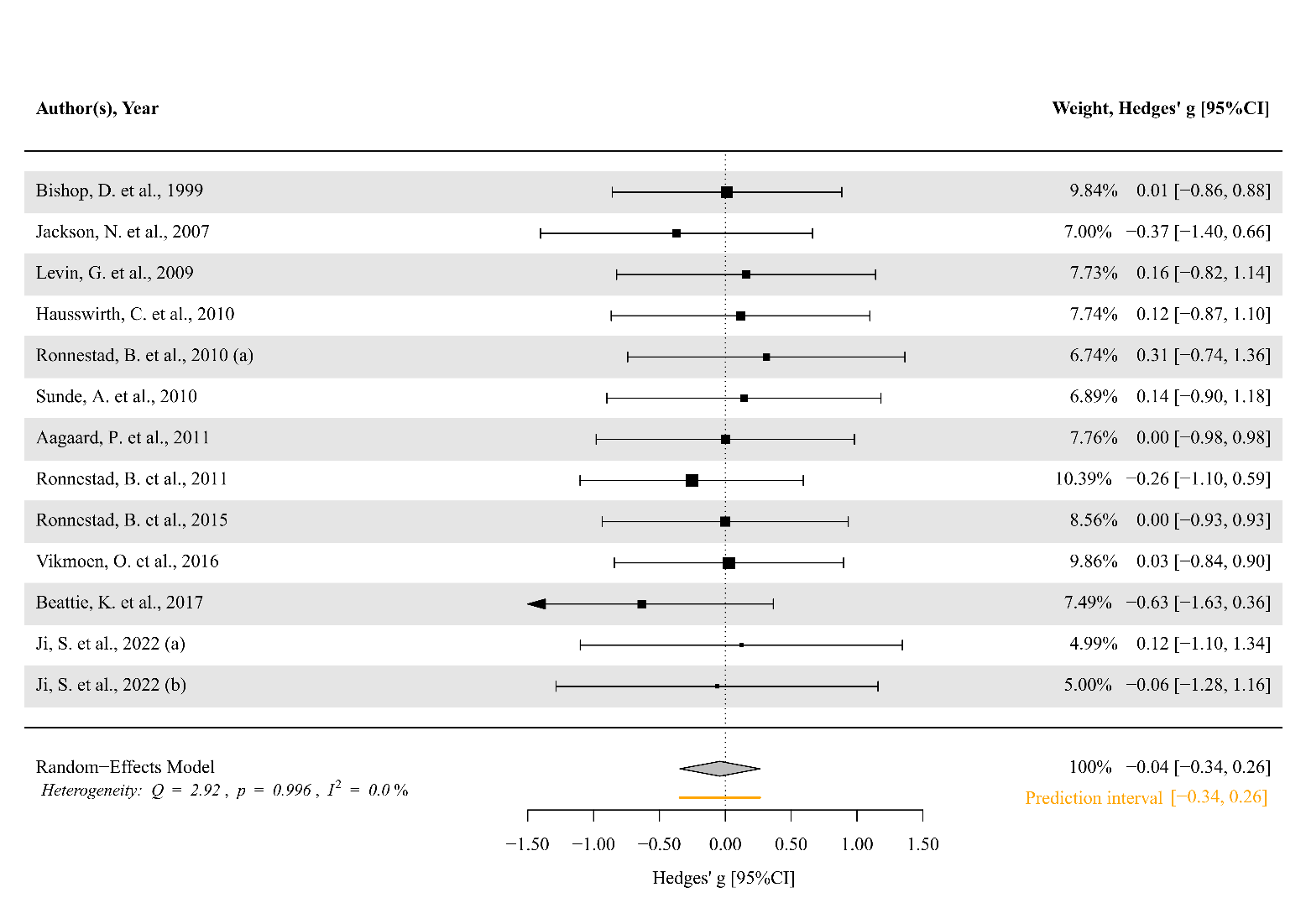


The black squares indicate the mean effect size observed for each study, and their size reflects the study weight. The black lines represent 95% confidence intervals. The diamond represents the pooled effect size (Hedges' g) with its 95% confidence interval. The orange line represents the prediction interval. A positive effect size represents a beneficial effect, while a negative effect size represents a detrimental effect.

**Fig. SM3** Forest plot of the effect of heavy strength training on power related to VO_2_max.


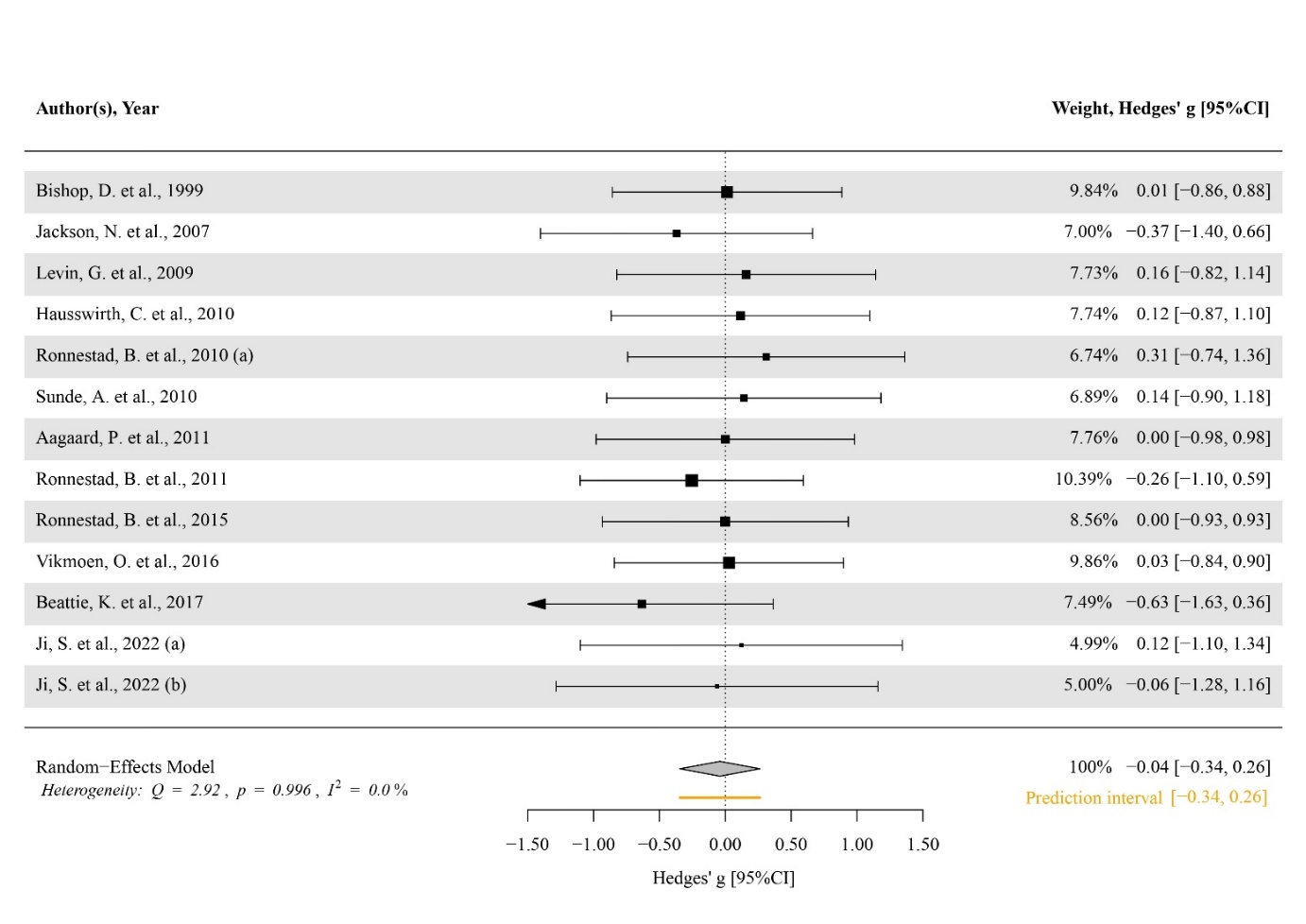


The black squares indicate the mean effect size observed for each study, and their size reflects the study weight. The black lines represent 95% confidence intervals. The diamond represents the pooled effect size (Hedges' g) with its 95% confidence interval. The orange line represents the prediction interval. A positive effect size represents a beneficial effect, while a negative effect size represents a detrimental effect.

**Fig. SM4** Forest plot of the effect of heavy strength training on maximal metabolic steady state.


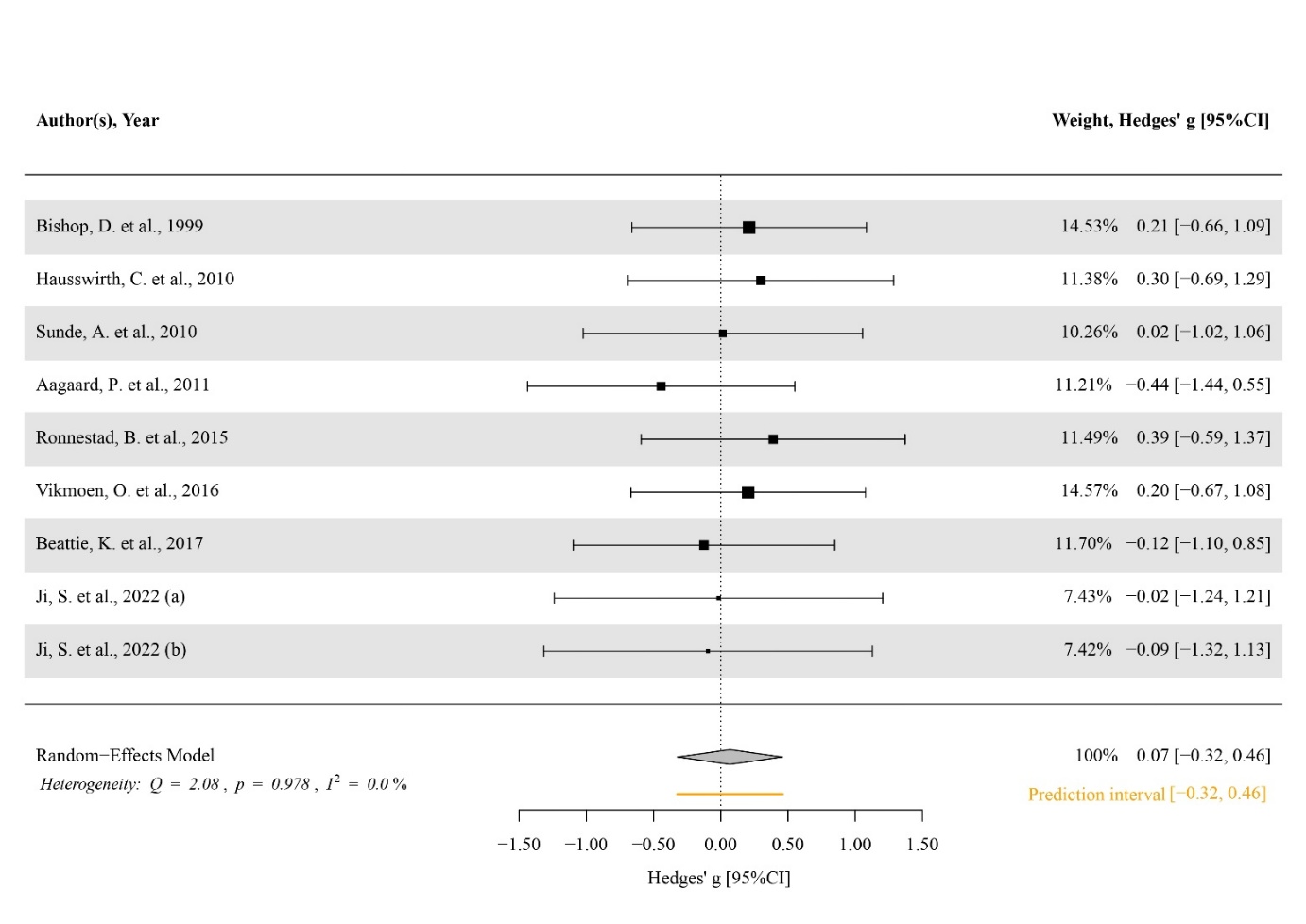


The black squares indicate the mean effect size observed for each study, and their size reflects the study weight. The black lines represent 95% confidence intervals. The diamond represents the pooled effect size (Hedges' g) with its 95% confidence interval. The orange line represents the prediction interval. A positive effect size represents a beneficial effect, while a negative effect size represents a detrimental effect.

**Fig. SM5** Forest plot of the effect of heavy strength training on anaerobic capacity.
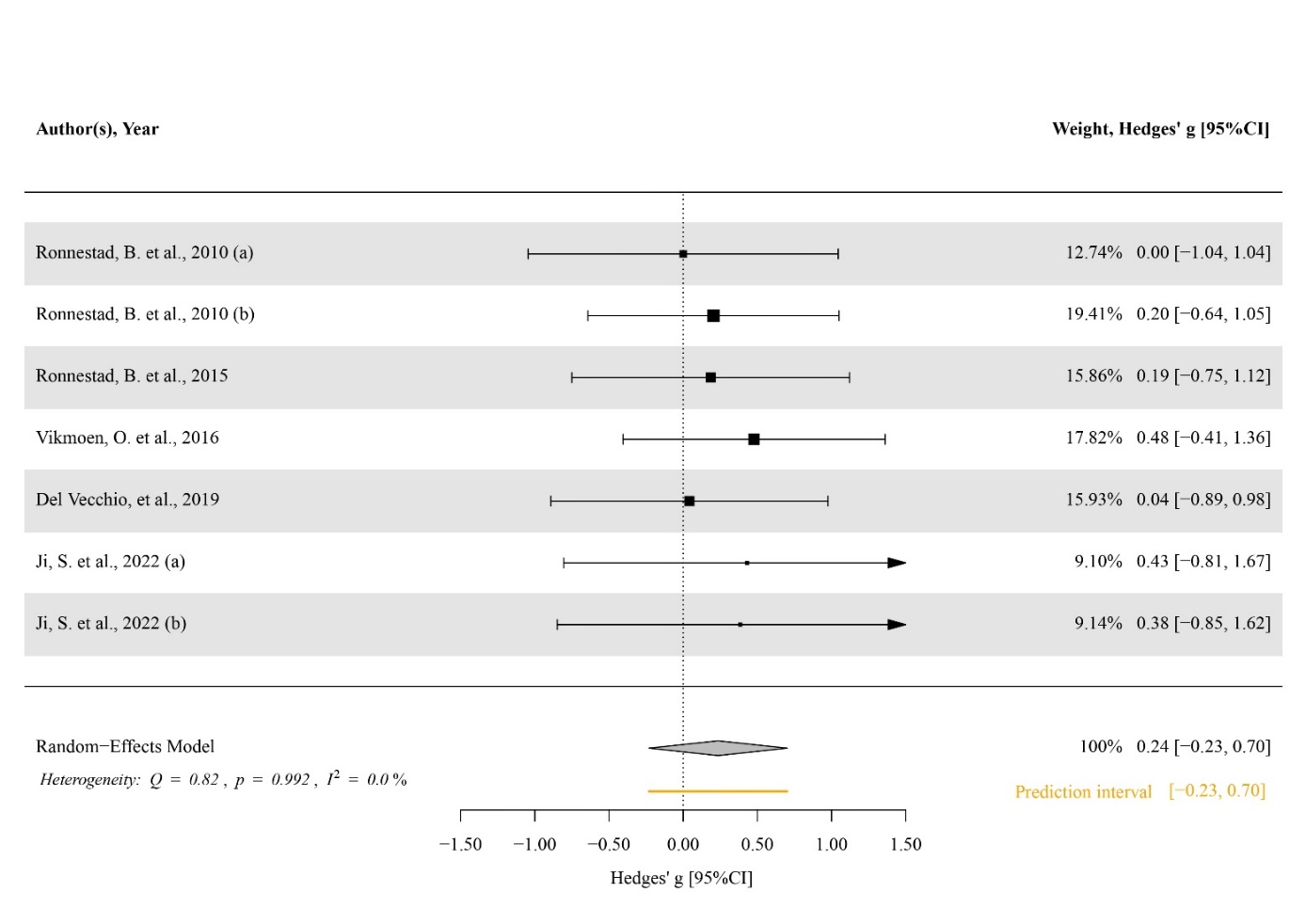
The black squares indicate the mean effect size observed for each study, and their size reflects the study weight. The black lines represent 95% confidence intervals. The diamond represents the pooled effect size (Hedges' g) with its 95% confidence interval. The orange line represents the prediction interval. A positive effect size represents a beneficial effect, while a negative effect size represents a detrimental effect.

**References**

Aagaard P, Andersen JL, Bennekou M, et al (2011) Effects of resistance training on endurance capacity and muscle fiber composition in young top-level cyclists. Scand J Med Sci Sports 21:. https://doi.org/10.1111/j.1600-0838.2010.01283.x

Bastiaans J, Diemen A, Veneberg T, Jeukendrup A (2001) The effects of replacing a portion of endurance training by explosive strength training on performance in trained cyclists. Eur J Appl Physiol 86:79–84. https://doi.org/10.1007/s004210100507

Beattie K, Carson BP, Lyons M, Kenny IC (2017) The Effect of Maximal- and Explosive-Strength Training on Performance Indicators in Cyclists. Int J Sports Physiol Perform 12:470–480. https://doi.org/10.1123/ijspp.2016-0015

Bishop D, Jenkins DG, Mackinnon LT, et al (1999) The effects of strength training on endurance performance and muscle characteristics. Med Sci Sports Exerc 31:886–891. https://doi.org/10.1097/00005768-199906000-00018

Bláfoss R, Rikardo J, Andersen A, et al (2022) Effects of Resistance Training Cessation on Cycling Performance in Well-Trained Cyclists: An Exploratory Study. J Strength Cond Res 36:796–804. https://doi.org/10.1519/JSC.0000000000004204

Del Vecchio L, Stanton R, Reaburn P, et al (2019) Effects of Combined Strength and Sprint Training on Lean Mass, Strength, Power, and Sprint Performance in Masters Road Cyclists. J Strength Cond Res 33:66–79. https://doi.org/10.1519/JSC.0000000000001960

Gil-Cabrera J, Valenzuela PL, Alejo LB, et al (2021) Traditional versus optimum power load training in professional cyclists: A randomized controlled trial. Int J Sports Physiol Perform 16:496–503. https://doi.org/10.1123/IJSPP.2020-0130

Hansen EA, Raastad T, Hallén J (2007) Strength training reduces freely chosen pedal rate during submaximal cycling. Eur J Appl Physiol 101:419–426. https://doi.org/10.1007/s00421-007-0515-7

Hansen EA, Rønnestad BR, Vegge G, Raastad T (2012) Cyclists’ Improvement of Pedaling Efficacy and Performance After Heavy Strength Training. Int J Sports Physiol Perform 7:313–321. https://doi.org/10.1123/ijspp.7.4.313

Hausswirth C, Argentin S, Bieuzen F, et al (2010) Endurance and strength training effects on physiological and muscular parameters during prolonged cycling. Journal of Electromyography and Kinesiology 20:330–339. https://doi.org/10.1016/j.jelekin.2009.04.008

Jackson NP, Hickey MS, Reiser RF (2007) High resistance/low repetition vs. low resistance/high repetition training: effects on performance of trained cyclists. J Strength Cond Res 21:289–95. https://doi.org/10.1519/R-18465.1

Ji S, Donath L, Wahl P (2022) Effects of Alternating Unilateral vs. Bilateral Resistance Training on Sprint and Endurance Cycling Performance in Trained Endurance Athletes: A 3-Armed, Randomized, Controlled, Pilot Trial. J Strength Cond Res 36:3280–3289. https://doi.org/10.1519/JSC.0000000000004105

Jones TW, Eddens L, Kupusarevic J, et al (2022) Effects of Cycling Intensity on Acute Signaling Adaptations to 8-weeks Concurrent Training in Trained Cyclists. Front Physiol 13:. https://doi.org/10.3389/fphys.2022.852595

Koninckx E, Leemputte M Van, Hespel P (2010) Effect of isokinetic cycling versus weight training on maximal power output and endurance performance in cycling. Eur J Appl Physiol 109:699–708. https://doi.org/10.1007/s00421-010-1407-9

Kristoffersen M, Sandbakk Ø, Rønnestad BR, Gundersen H (2019) Comparison of short-sprint and heavy strength training on cycling performance. Front Physiol 10:. https://doi.org/10.3389/fphys.2019.01132

Levin GT, Mcguigan MR, Laursen PB (2009) Effect of Concurrent Resistance and Endurance Training on Physiologic and Performance Parameters of Well-Trained Endurance Cyclists. J Strength Cond Res 23:2280–2286. https://doi.org/10.1519/JSC.0b013e3181b990c2

Louis J, Hausswirth C, Easthope C, Brisswalter J (2012) Strength training improves cycling efficiency in master endurance athletes. Eur J Appl Physiol 112:631–640. https://doi.org/10.1007/s00421-011-2013-1

Luckin-Baldwin KM, Badenhorst CE, Cripps AJ, et al (2021) Strength Training Improves Exercise Economy in Triathletes During a Simulated Triathlon. Int J Sports Physiol Perform 16:663–673. https://doi.org/10.1123/ijspp.2020-0170

McNamara JM, Stearne DJ (2013) Effect of Concurrent Training, Flexible Nonlinear Periodization, and Maximal-Effort Cycling on Strength and Power. J Strength Cond Res 27:1463–1470. https://doi.org/10.1519/JSC.0b013e318274f343

Minahan C, Wood C (2008) Strength training improves supramaximal cycling but not anaerobic capacity. Eur J Appl Physiol 102:659–666. https://doi.org/10.1007/s00421-007-0641-2

Montalvo-Pérez A, Alejo LB, Valenzuela PL, et al (2021) Traditional Versus Velocity-Based Resistance Training in Competitive Female Cyclists: A Randomized Controlled Trial. Front Physiol 12:. https://doi.org/10.3389/fphys.2021.586113

Moysi JS, Garcia CD, Izquierdo JC, Calbet JAL (1998) Improvement of cycling efficiency after strength training. Archivos de Medicina del Deporte 15

Paton CD, Hopkins WG (2005) Combining Explosive and High-Resistance Training Improves Performance in Competitive Cyclists. The Journal of Strength and Conditioning Research 19:826. https://doi.org/10.1519/R-16334.1

Psilander N, Frank P, Flockhart M, Sahlin K (2015) Adding strength to endurance training does not enhance aerobic capacity in cyclists. Scand J Med Sci Sports 25:e353–e359. https://doi.org/10.1111/sms.12338

Rønnestad BR, Hansen EA, Raastad T (2011) Strength training improves 5-min all-out performance following 185 min of cycling. Scand J Med Sci Sports 21:250–259. https://doi.org/10.1111/j.1600-0838.2009.01035.x

Rønnestad BR, Hansen EA, Raastad T (2012) Strength Training Affects Tendon Cross-Sectional Area and Freely Chosen Cadence Differently in Noncyclists and Well-Trained Cyclists. J Strength Cond Res 26:158–166. https://doi.org/10.1519/JSC.0b013e318218dd94

Rønnestad BR, Hansen EA, Raastad T (2010a) In-season strength maintenance training increases well-trained cyclists’ performance. Eur J Appl Physiol 110:1269–1282. https://doi.org/10.1007/s00421-010-1622-4

Rønnestad BR, Hansen EA, Raastad T (2010b) Effect of heavy strength training on thigh muscle cross-sectional area, performance determinants, and performance in well-trained cyclists. Eur J Appl Physiol 108:965–975. https://doi.org/10.1007/s00421-009-1307-z

Rønnestad BR, Hansen J, Hollan I, et al (2016) Impairment of performance variables after in-season strength-Training cessation in elite cyclists. Int J Sports Physiol Perform 11:727–735. https://doi.org/10.1123/ijspp.2015-0372

Rønnestad BR, Hansen J, Hollan I, Ellefsen S (2015) Strength training improves performance and pedaling characteristics in elite cyclists. Scand J Med Sci Sports 25:e89–e98. https://doi.org/10.1111/sms.12257

Rønnestad BR, Hansen J, Nygaard H (2017) 10 weeks of heavy strength training improves performance-related measurements in elite cyclists. J Sports Sci 35:1435–1441. https://doi.org/10.1080/02640414.2016.1215499

Silva MH, Andre Barbosa De Lira C, Steele J, et al (2022) Cycle ergometer training and resistance training similarly increase muscle strength in trained men. J Sports Sci 40:583–590. https://doi.org/10.1080/02640414.2021.2005282

Sitko S, López-Laval I, Cirer-Sastre R (2024) Influence of Conventional Resistance Training Compared to Core Exercises on Road Cycling Power Output. Cureus. https://doi.org/10.7759/cureus.59371

Sunde A, Støren Ø, Bjerkaas M, et al (2010) Maximal Strength Training Improves Cycling Economy in Competitive Cyclists. J Strength Cond Res 24:2157–2165. https://doi.org/10.1519/JSC.0b013e3181aeb16a

Valenzuela PL, Gil-Cabrera J, Talavera E, et al (2021) On- versus off-bike power training in professional cyclists: A randomized controlled trial. Int J Sports Physiol Perform 16:674–681. https://doi.org/10.1123/ijspp.2020-0305

Vikmoen O, Ellefsen S, Trøen, et al (2016) Strength training improves cycling performance, fractional utilization of VO2max and cycling economy in female cyclists. Scand J Med Sci Sports 26:384–396. https://doi.org/10.1111/sms.12468

Vikmoen O, Rønnestad BR, Ellefsen S, Raastad T (2017) Heavy strength training improves running and cycling performance following prolonged submaximal work in well-trained female athletes. Physiol Rep 5:. https://doi.org/10.14814/phy2.13149

Zoladz JA, Szkutnik Z, Majerczak J, et al (2012) Isometric strength training lowers the O2 cost of cycling during moderate-intensity exercise. Eur J Appl Physiol 112:4151–4161. https://doi.org/10.1007/s00421-012-2405-x
